# Identification and Analysis of Unstructured, Linear B-Cell Epitopes in SARS-CoV-2 Virion Proteins for Vaccine Development

**DOI:** 10.3390/vaccines8030397

**Published:** 2020-07-20

**Authors:** Andrés Corral-Lugo, Mireia López-Siles, Daniel López, Michael J. McConnell, Antonio J. Martin-Galiano

**Affiliations:** 1 Intrahospital Infections Unit, National Centre for Microbiology, Instituto de Salud Carlos III (ISCIII), 28220 Madrid, Spain; acorral@isciii.es (A.C.-L.); mireia.lopez@isciii.es (M.L.-S.); 2 Immune Presentation and Regulation Unit, Instituto de Salud Carlos III, 28220 Madrid, Spain; dlopez@isciii.es

**Keywords:** coronavirus, COVID-19, epitope prediction, epitope switch, immunoinformatics, unfolded protein response, SARS-CoV

## Abstract

The efficacy of SARS-CoV-2 nucleic acid-based vaccines may be limited by proteolysis of the translated product due to anomalous protein folding. This may be the case for vaccines employing linear SARS-CoV-2 B-cell epitopes identified in previous studies since most of them participate in secondary structure formation. In contrast, we have employed a consensus of predictors for epitopic zones plus a structural filter for identifying 20 unstructured B-cell epitope-containing loops (uBCELs) in S, M, and N proteins. Phylogenetic comparison suggests epitope switching with respect to SARS-CoV in some of the identified uBCELs. Such events may be associated with the reported lack of serum cross-protection between the 2003 and 2019 pandemic strains. Incipient variability within a sample of 1639 SARS-CoV-2 isolates was also detected for 10 uBCELs which could cause vaccine failure. Intermediate stages of the putative epitope switch events were observed in bat coronaviruses in which additive mutational processes possibly facilitating evasion of the bat immune system appear to have taken place prior to transfer to humans. While there was some overlap between uBCELs and previously validated SARS-CoV B-cell epitopes, multiple uBCELs had not been identified in prior studies. Overall, these uBCELs may facilitate the development of biomedical products for SARS-CoV-2.

## 1. Introduction

In December 2019, Wuhan, China became the center of an outbreak of febrile respiratory illness due to a new type of coronavirus [1]. Genome sequencing showed genetic similarities to other coronaviruses found in humans [2], particularly with Severe Acute Respiratory Syndrome Coronavirus (SARS-CoV), resulting in the designation of this virus as SARS-CoV-2 by the World Health Organization (WHO) [3]. The number of globally confirmed cases according to the WHO reached 9,129,146 and 473,797 deaths by 24 June 2020.

Human coronaviruses, first characterized in the 1960s, are responsible for upper respiratory tract infections [4], and can infect both human and animal hosts [4,5]. In addition to the current pandemic, coronaviruses have previously caused two outbreaks involving respiratory infections with great repercussions. In 2003, SARS-CoV affected more than 8000 people in 25 countries across five continents [6] and, in 2012, the Middle East Respiratory Syndrome coronavirus infected more than 1000 patients with a mortality rate of more than 35% [7].

Similarly to other coronaviruses, SARS-CoV-2 has a ~30 kb single-stranded, positive-sense RNA genome containing genes that encodes homologs for at least four main structural proteins of the viral particle: spike (S), envelope (E), membrane (M), and nucleocapsid (N) proteins [8]. Functional and immunological properties of these proteins have been well-studied in SARS-CoV since 2003. For instance, the S protein is a glycoprotein that forms homotrimers and consists of two functional subunits responsible for binding to the host cell receptor (S_1_ subunit) and for the fusion of the viral and cellular membranes (S_2_ subunit), as also confirmed in SARS-CoV-2 [9]. The distal S_1_ subunit contains the receptor-binding domain (S_1B_) necessary for attachment to the ACE2 receptor and entry into type II pneumocytes and other host cells. Active immunization with full-length and truncated S protein [10], S protein peptides [11], and chimeric versions of the S protein [12] has been characterized for SARS-CoV. DNA constructs encoding the S protein produce neutralizing antibodies against the virus [13,14]. Therefore, the S protein of both SARS-CoV and SARS-CoV-2 is a major target for vaccine development. The small multifunctional E protein is important for pathogenesis and different steps in the virus life cycle (assembly, budding, and envelope formation). Although only a small portion of E is incorporated into the virion, it is abundantly expressed inside the infected cell [15]. The E protein is recognized by SARS-CoV convalescent sera [16]; however, limited information is available regarding its antigenic properties.

The transmembrane M glycoprotein is the most abundant structural protein in the mature coronavirus virion and has been suggested to play a major role in envelope formation in SARS-CoV [17]. IgM and IgG antibodies against the M protein are present in sera from patients with SARS-CoV [18], and high titers of these antibodies are elicited in rabbits immunized with the N-terminal region of the protein [10,19,20]. The N protein contains an amino-terminal RNA binding domain and a carboxyl-terminal dimerization domain. This protein is involved in envelope formation, regulation of viral RNA synthesis, packaging of viral RNA, and may play an important role in overcoming host defense by suppressing RNA interference mechanisms [21,22]. The SARS-CoV N protein is highly immunogenic and antigenic sites have been described throughout the entire sequence [18,23,24]. Moreover, N protein is an early diagnostic marker for SARS-CoV because it is detectable in clinical samples as early as one day after the onset of symptoms [25].

It is clear that the fight against SARS-CoV-2 requires a number of clinical approaches that are not currently available. The identification of viral B-cell epitopes, which are the key elements that trigger the protective humoral immune response, can facilitate the design and development of vaccines, rapid diagnostic tests, and antibody-based therapeutics. In addition, characterization of such epitopes can contribute to our understanding of mutational changes that affect the ability of the immune response to provide cross protection against related viruses. Multiple immunoinformatic approaches have been developed for the prediction of B-cell epitopes based on different criteria that aim to capture the intrinsic complexity of the binding between the antigenic epitope and the antibody paratope [26]. However, given that the sensitivity for detection of linear epitopes using computational approaches has been estimated to be around 60% [27], the integration of several methods may identify physiologically relevant B-cell epitopes more accurately. In addition to the discontinuous and structured nature of many B-cell epitopes [28], numerous examples of linear epitopes located in loops and inducing immunoprotective humoral responses and their recognition by antibodies in laboratory assays have been described for relevant viral pathogens [29,30,31], highlighting their potential utility in biomedical applications.

Given the inherent difficulty of experimentally mapping B-cell epitopes [32], several studies have used computational approaches to predict B-cell epitopes from SARS-CoV [13,33,34,35,36,37]. Multiple B-cell epitopes from SARS-CoV have been experimentally validated since 2003 [38] and included in the Immune Epitope Database (IEDB), a central repository that stores, catalogs, and assists in the prediction and analysis of epitopes [39]. In contrast to SARS-CoV, there is still relatively little information available regarding B-cell epitopes from SARS-CoV-2. Nevertheless, both in vitro [40] and in silico analyses of proteins S [41,42,43,44,45], M and N [46] have been conducted to determine sequence variation, antigenic regions, and targets of the immune responses in SARS-CoV-2.

A myriad of vaccine initiatives for SARS-CoV-2 based on different technologies are currently being undertaken [47]. One of the most promising approaches is the utilization of DNA vaccines coding for viral epitopic regions from different antigens. However, these highly-complex chimeric proteins include incomplete sections of protein folds that may adopt aberrant structural arrangements when present in isolation. This situation may trigger the cellular unfolded protein response (UPR) in host cells. During the UPR, hydrophobic residues normally buried in the context of the full-length antigen may be exposed and sensed in the endoplasmic reticulum by the GRP78 chaperone, which labels the anomalous protein for cytoplasm back-translocation and ubiquitin-mediated proteolysis [48]. The presence of misfolded sequences in multiepitope vaccines may therefore result in antigen degradation, potentially decreasing the ability of the antigen to induce a robust immune response.

To address this problem, and in contrast to recent reports predicting B-cell epitopes in SARS-CoV-2, the present study focuses on the identification and characterization of unstructured B-cell epitope-containing loops (uBCELs) of virion proteins in order to avoid triggering the UPR. These sequences may therefore be ideally suited for the development of diagnostic, therapeutic, and preventive technologies for SARS-CoV-2 infection, particularly multiepitope vaccines.

## 2. Materials and Methods

SARS-CoV and SARS-CoV-2 proteins utilized in this study were those in the reference proteome provided by NCBI entries NC_004718 [49] and NC_045512 [50], respectively. For a detailed bibliography of the databases and methodology used, see Supplementary Table S1.

### 2.1. Identification of uBCELs

Linear B-cell epitopes (BCEs) were predicted by the SVM2 model of AAPPred with a score ≥ 0; ABCPred with a score ≥ 0.8; Bepipred applying a score of ≥0.35; Bepipred 2.0, applying a score of ≥0.5; Kolaskar’s antigenicity applying a score threshold ≥ 0.988; LBEEP using window length of 15 residues and a score of 0.7; and SVMtrip, applying a score of ≥0.35. In addition, an eighth method consisted of collectively analyzing the average value resultant from six physicochemical predictors related to B-cell antigenicity: Emini accessibility, Janin surface exposure, Karplus & Schulz flexibility, Parker hydrophilicity, Pellenquer turns and Ponnuswamy polarity, applying an average value of 1.2 as threshold.

Sequence spans of ≥6 residues (including a maximum of 1 internal non-predicted residue), supported by at least four out of eight algorithms were considered as candidate linear B-cell epitopes. These preliminary epitopes were filtered by resolved or predicted secondary protein structure, and only those with ≥6 residues in non-folded regions were denoted unstructured BCEs (uBCEs). Finally, the entire unstructured loop containing each uBCE was considered as uBCELs.

Antigenicity was predicted using the iLBE (http://kurata14.bio.kyutech.ac.jp/iLBE/) and VaxiJen 2.0 (http://www.ddg-pharmfac.net/vaxijen/VaxiJen/VaxiJen.html) servers.

### 2.2. Structural and Accessory Analysis of Protein Antigens

Secondary structure analysis was carried out using available three-dimensional structures that covered most of the S and N proteins: 6M3M (unpublished) (41–174 residues, 100% identical) and 2CJR [51] (250–360 residues, 95% identical) PDB entries for N protein; and the 6VXX_A entry (14–1211 residues, 100% identical) for S protein [9]. For E and M proteins, structures were not available and models were deemed low-quality by Swiss Model curators (https://swissmodel.expasy.org/repository/species/2697049), and therefore were not considered. For regions and proteins without available experimental structural information, secondary structure was predicted by PSI-PRED 4.0. Protein domains were identified with Pfam 32.0 applying gathering thresholds; transmembrane helices (TMHs) were predicted by Phobius; signal peptides were predicted by SignalP 5.0 applying the Eukarya model; coiled-coil regions were predicted by COILS using the MTIDK matrix, and applying a threshold of 0.5 with 14, 21 and 28 residue windows; disordered regions were predicted by the long model of IUPred2, using 0.5 as threshold, and by DISOPRED 3.1, finally considering disordered regions as those with ≥5 residues predicted by at least one of these methods.

### 2.3. Phylogenetic Analyses

All non-redundant coronavirus homologs of the Identical Protein Groups database for the four selected proteins were detected by BLAST using the reference S, E, M, and N SARS-CoV-2 sequences as queries. A 70% identity threshold, 90% alignment length coverage, and E value < 10^−5^ were applied. Partial hit sequences and those containing ‘X’s were removed. Updated SARS-CoV-2 protein sequences were obtained from the coding region files provided by the NCBI. Then, the dataset was reduced on a 95% identity and a 95% reciprocal protein length coverage basis using CD-HIT. Alignments were carried out with Clustal Omega. The evolutionary history was calculated by the Neighbor-Joining method using Mega 7. Branch lengths were proportional to the tree evolutionary distances calculated using the Poisson correction method. The rate variation among sites was modeled with a gamma distribution applying a shape parameter of 1. Phylogeny was tested by 1000 bootstrap replicates. SARS-CoV and SARS-CoV-2 clades were identified through the location of their respective reference sequences.

Sequences from SARS-CoV and SARS-CoV-2 clades were re-aligned with Clustal Omega and SDPs identified with SDPpred applying 1000 shuffles and a Bernoulli estimator cutoff of 0.

### 2.4. Epitope Collection in SARS-CoV-2

For SARS-CoV, experimentally determined and predicted epitopes of S, E, M, and N proteins were obtained from the IEDB. The following filters were applied: “linear epitope”, “B-cell assay”, “severe acute respiratory syndrome-related coronavirus organism (ID: 694009, SARS)”, “severe acute respiratory syndrome disease data (ID: DOID:2945, SARS)”, “any MHC restriction”, and “any host”. The published studies describing these epitopes were identified and screened in order to include only epitopes that had been validated as being able to stimulate an antibody response in experimental models. Published epitopes of SARS-CoV-2 were identified based on the bibliography available in PubMed up to 31 March 2020. The following keywords were used: “S, E, M or N protein”, “epitope”, “SARS-CoV-2 or COVID-19”. Only those epitopes with less than 100 amino acids were considered.

### 2.5. Statistical Analyses

To compare length and percentage of residues in unstructured regions of previously reported SARS-CoV-2 epitopes, the Shapiro–Wilk test was firstly used to assess data normality and Levene’s test used to assess for equality of variances. Given the non-normal distribution and the lack of homoscedasticity, the non-parametric Kruskal–Wallis test was applied. Significance of the occurrence of SDPs and indels in uBCELs with respect to the rest of the protein sequence was assessed by the chi-square test. Statistical analyses were conducted via the SPSS 15.0 statistical package for Windows (IBM Corp., Armonk, NY, USA). Significance levels were established for *p*-values ≤ 0.05.

## 3. Results

### 3.1. SARS-CoV-2 Epitope Catalogue

**Figure 1:**
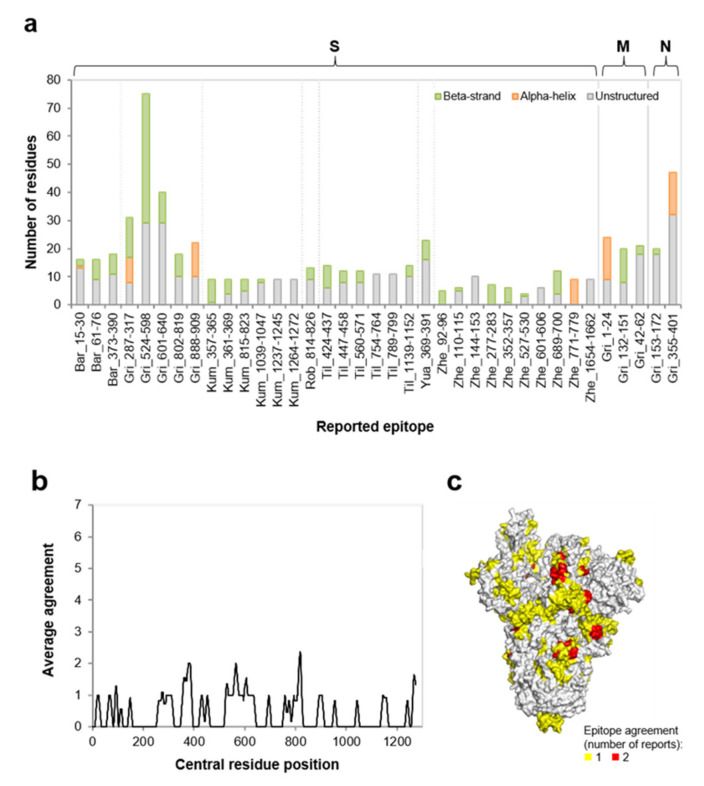
Characteristics of B-cell epitopes on SARS-CoV-2 proteins reported in the literature. (**a**) length and predicted secondary structure content of reported epitopes (*n* = 37) by protein and study. Report prefixes: Bar [44], Gri [46], Kum [41], Rob [45], Til [42], Yua [40], and Zhe [43]. Experimentally reported or predicted alpha-helices and beta-strands were considered; (**b**) average agreement of reported epitopes for S protein between the seven studies. The average value of a sliding window of 11 residues was calculated and this value assigned to the residue in the central position; (**c**) surface view of the S protein three-dimensional structure showing color-ranked epitope agreement.

The four SARS-CoV-2 structural proteins were predicted as antigens by the iLBE and VaxiJen methods (data not shown). A screen of SARS-CoV-2 linear B-cell epitopes reported in the literature up to Apr 08th 2020 was performed. A total of 37 epitopes from seven studies were included (Supplementary Table S2). Of these, more than 85% were located in the S protein (*n* = 32), with the remaining in the N (*n* = 2) or M (*n* = 3) proteins (Figure 1a). The predominance of epitopes identified in the S protein may be explained by bias of the included studies towards utilization of only the S protein and the fact that the S protein may be more suited for epitope identification given its length, structure, and higher solvent exposure in the virus capsid. To the best of our knowledge, no linear B-cell epitopes have been reported for the SARS-CoV-2 E protein.

Up to ten epitopes were identified in the included studies, with a median length of 12 amino acids. Epitopes ranged from five to 75 residues, with different studies demonstrating significant variations when their epitope lengths were compared (*p* < 0.001), indicating a mixture of pure epitopes and wide epitopic zones. Thirty protein epitopes (81.1%) from these studies showed significant overlap of their sequence (between 10 and 100%) with protein structured elements (either alpha-helices or beta-strands), with no significant difference between studies included in the analysis (*p* > 0.928).

For the S protein, the only protein analyzed in more than one study, we further characterized the agreement of reported epitopes for the seven articles included in the analysis. This meta-analysis revealed that residues were identified as contributors of predicted epitope elements by, at most, two independent studies (Figure 1b). The three-dimensional positioning of these residues further indicated that they do not aggregate either into domains or regions, but are dispersed over the entirety of the S protein structure (Figure 1c). Together, these analyses indicate that there is little agreement between studies regarding the linear SARS-CoV-2 B-cell epitopes in the published literature. Furthermore, the proposed epitopes identified in these studies tend to include portions of structured elements, which may misfold and trigger the UPR when expressed in the endoplasmic reticulum.

### 3.2. Unstructured Epitope Selection to Design Antigenic Peptides and Chimera Proteins

**Figure 2:**
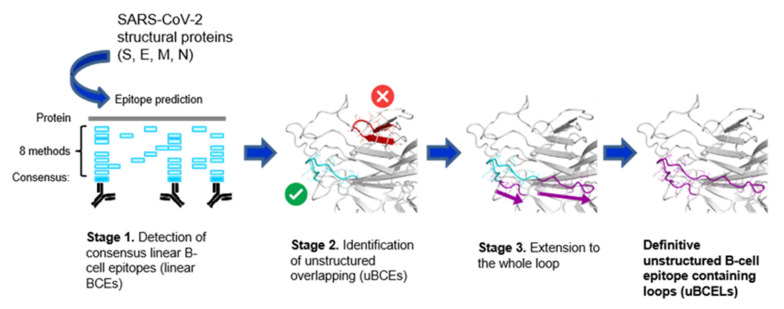
In silico pipeline for the identification of uBCELs. A schematic three-stage flowchart to define uBCELs in SARS-CoV-2 virion proteins is shown. First, eight bioinformatics methods were collectively utilized to locate, by consensus, the linear B-cell epitopes in S, E, M, and N proteins. Then, those B-cell epitopes that co-located to regular secondary structure elements, either alpha-helices or beta-strands, were rejected. Finally, the location of B-cell epitopes that satisfied the former criteria was extended to cover the complete loop.

A plausible strategy for generating antibodies against SARS-CoV-2 using peptide or multipeptide antigens involves the utilization of unstructured sections harboring B-cell epitopes from the four virion proteins. Loops containing linear B-cell epitopes were identified in these four proteins using a three-stage computational pipeline (Figure 2). In a first step, and given the low sensitivity of the detection of these epitopes, a consensus of eight available prediction methods was designed (see Methods). This inclusive approach is expected to cover technical predictive aspects as well as physicochemical nuances of these types of epitopes. Second, epitopes satisfying the initial criteria were placed into structural context. To avoid potentially triggering the UPR by misfolded sequences, only predicted linear BCEs located in loops, i.e., uBCEs, were selected. All uBCEs contained at least six unstructured residues, since most B-cell linear epitopes fall within the 6–10 residue range [52]. A total of 21 unstructured B-cell epitopes were identified using this approach. These were extended to the whole disordered region to render the corresponding uBCELs (Table 1). Eleven uBCELs were detected in the S, one in M, and eight in N proteins for a total of 20 uBCELs since two epitopes were located in the same loop. In addition, a structured exception involving a B-cell epitope in an alpha-helical section in the E protein (BCEH-E1) was also considered (Table 1).

**Figure 3:**
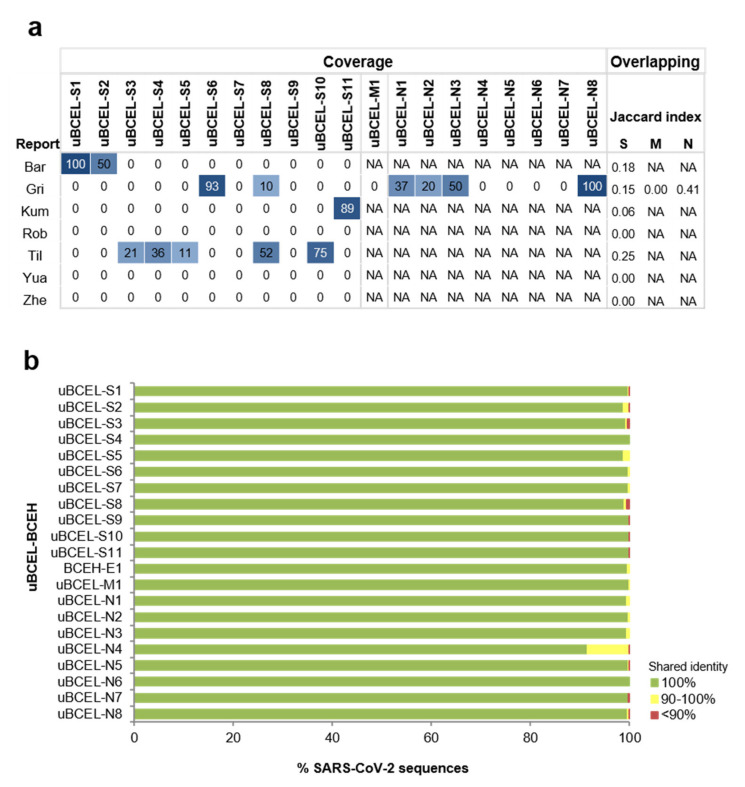
Characteristics of uBCELs. (**a**) heatmap of uBCELs coverage by protein and study. Coverage is also ranked by color intensity. Report prefixes: Bar [44], Gri [46], Kum [41], Rob [45], Til [42], Yua [40], and Zhe [43]. Not applicable (NA) labels are indicated for reports that did not propose epitopes for M or N proteins. Jaccard indices for residue overlapping are indicated on right; (**b**) conservation of uBCELs in SARS-CoV-2 sequenced genomes. Genomes were acquired applying the “COVID-19” organism in the NCBI nucleotide database (Last accession: 1 May 2020).

Comparison of these uBCELs to reported SARS-CoV-2 epitopes through a residue coverage matrix showed that overlap between uBCELs and previously identified epitopes was poor or null (Figure 3a). Jaccard indices ≤ 0.25, i.e., overlapping residues were below a quarter of the total, were obtained in all cases except for the N-protein when compared to the dataset from Grifoni and colleagues [46] that reached 0.41.

It is of interest to assess how uBCELs have evolved between SARS-CoV and SARS-CoV-2 with respect to the rest of the protein. In the S protein, uBCELs covered 15.7% of the protein sequence (excluding the 15mer signal peptide). However, they were significantly enriched (36.7%) in insertions and specificity determining positions (SDPs)—those positions with conserved exclusive residues in each clade, which greatly determine phylogenetic tree structure [53] (*p* < 0.0001). This may suggest higher evolutionary dynamics in these sequences, perhaps due in part to the selective pressure for epitope switching.

In contrast, when uBCEL sequence variability was inspected within an updated sampling (Last accession: 1 May 2020) of 1639 SARS-CoV-2 isolates with complete genome sequences, only four uBCELs demonstrated residue changes in more than 1% of isolates (Figure 3b and Table 2). Among these, the I68- deletion in uBCEL-S2, V483A in uBCEL-S5, and the S197L, R203K-G204R, and T205I in uBCEL-N4 variants were observed in 10 or more isolates. All these predominant lineages were detected for the first time in isolates obtained between 29 January 2020 and 15 March 2020. In particular, the R203K-G204R variant in N was particularly prevalent since it was detected in samples involving nine countries from three continents.

### 3.3. S Protein uBCEL Analysis

**Figure 4:**
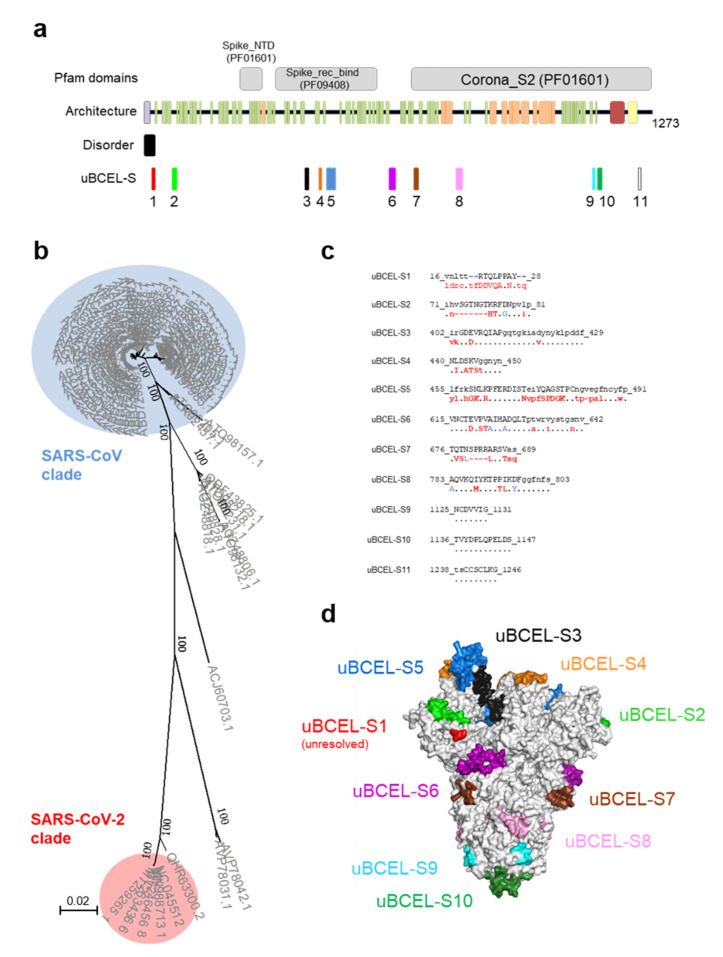
Architecture and analysis of predicted uBCELs on spike S protein. (**a**) a graphical depiction of S protein with Pfam domains, architecture, disorder and uBCELs localization. Color blocks: Pfam domains (grey), alpha-helices (orange), beta-strand (green), transmembrane helices (yellow), signal peptide (purple), coiled-coils (red) and disordered regions (black). In addition, each uBCEL is shown in its own color; (**b**) phylogenetic tree of 89 non-redundant coronavirus S sequences calculated by the Neighbor-Joining method. Bootstrap values of 100 are indicated; (**c**) SARS-CoV-2 uBCEL-S sequences (including deletions) and changes observed in relation to SARS-CoV S protein: capital letters indicate epitopes, residues conserved in ≥90% sequences (dots), changed to unique option (≥90%, red), ambiguous changes (two or more residue option in >10% sequences, blue), and deletions (dashes); (**d**) structural mapping of uBCELs on the surface view of the modeled S protein homotrimer. Each uBCEL is depicted in the same colors as in Figure 4a. uBCEL-S11 falls out of the resolved section of the protein and therefore is not shown.

The S protein is by far the largest and most complex antigenic polypeptide in the SARS-CoV-2 virion. This protein has two subunits, three predicted domains according to the Pfam database that detect independent protein sections through hidden Markov models, a signal peptide, one TMH, at least one coiled-coil region, and numerous alpha-helices and beta-strands (Figure 4a). Four of the eleven uBCELs harbor N-linked-glycosylation sites, a potentially important factor used to evade the immune response [54]. In addition, three uBCELs (-S3, -S4 and -S5) fell in the S1B domain (323–502 residues) and include six of the fourteen residues that directly interact with the host ACE2 receptor. The uBCEL-S1, -S2, -S4, -S5, and -S7 are among the most divergent regions (showing 4–22 changes and up to seven inserted residues) between SARS-CoV and SARS-CoV-2 (Figure 4b,c). These mutational hotspots affect the apical half of the protein (Figure 4d).

### 3.4. E Protein Epitope Analysis

**Figure 5:**
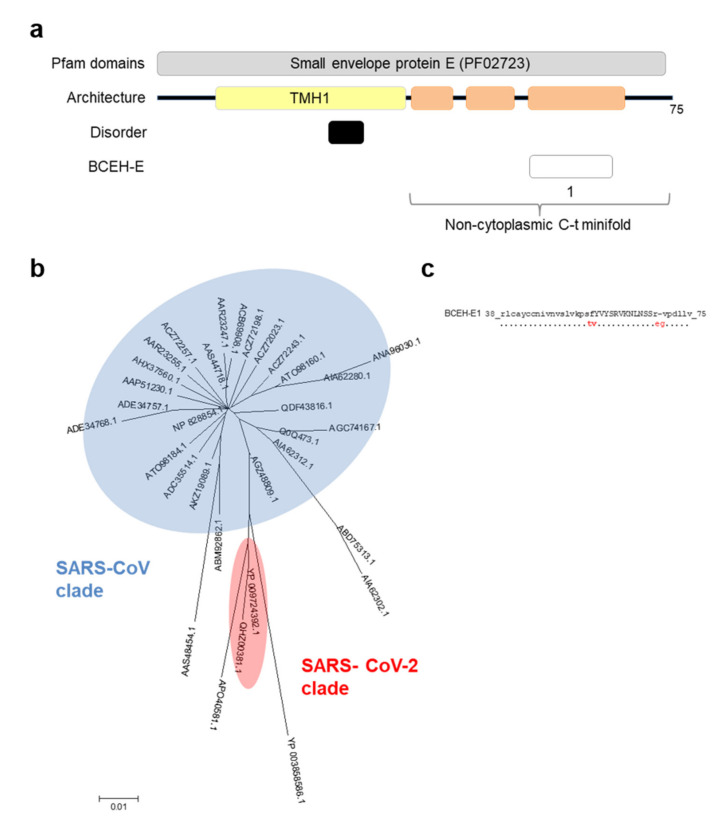
Architecture and analysis of BCEH of envelope E protein. (**a**) a graphical depiction of E protein with Pfam domains, architecture, disorder and BCEH localization. Color blocks: Pfam domains (grey), alpha-helices (orange), transmembrane helices (yellow), disordered regions (black), and BCEH (white); (**b**) phylogenetic tree of 32 non-redundant coronavirus E sequences calculated by the Neighbor-Joining method. No bootstrap value reached a value of 100; (**c**) SARS-CoV-2 BCEH-E sequence and changes observed in relation to SARS-CoV E protein: capital letters indicate epitopes, residues conserved ≥90% sequences (dots), changed to unique option (≥90%, red), deletions (dashes).

The E protein is a small polypeptide with a predicted TMH and 38 residues oriented toward the exterior of the virion (Figure 5a). Nevertheless, this region is predicted to contain three alpha-helices that may interact to produce a minifold. The additional absence of disorder strongly suggests that this section is completely folded. Within the 38-mer minifold, there is a 12-mer within the predicted alpha-helix 3 with high predicted antigenicity, BCEH-E1. This is the only structured exception included in our analyses. The SARS-CoV-2 minifold sequence only showed three changes and one deletion with respect to the SARS-CoV clade sequences, although all fall outside the epitope (Figure 5b,c).

### 3.5. M Protein Epitope Analysis

**Figure 6:**
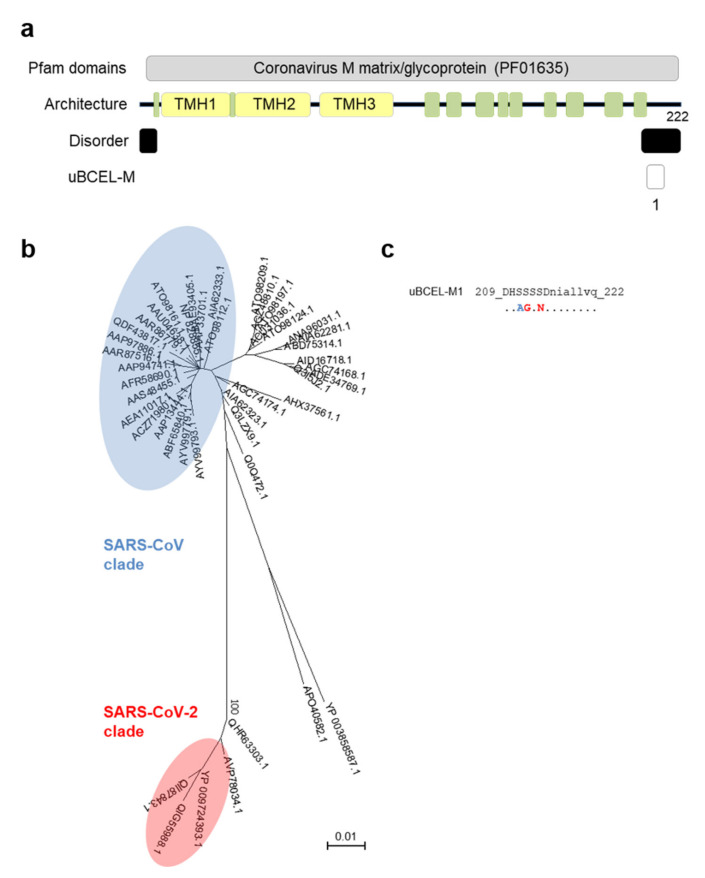
Architecture and analysis of uBCELs of matrix glycoprotein M. (**a**) a graphical depiction of M protein with Pfam domains, architecture, disorder, and uBCELs localization. Color blocks: beta-strand (green), transmembrane helices (yellow), disordered regions (black), and uBCELs (white); (**b**) phylogenetic tree of 44 non-redundant coronavirus M sequences calculated by the Neighbor-Joining method. Bootstrap values of 100 are indicated; (**c**) SARS-CoV-2 uBCEL-M sequence and changes observed in relation to SARS-CoV M protein: capital letters indicate epitopes, residues conserved ≥90% sequences (dots), changed to unique option (≥90%, red), ambiguous changes (two or more residue option in >10% sequences, blue).

The M protein is also a peripheral membrane protein, with 3 TMHs and a globular carboxyl-terminal section of 112 residues enriched in predicted beta-strands (Figure 6a). Although some loops in this region are evident, the protein is predicted to have low disorder. Only one uBCEL was detected, close to the C-terminus, with two conservative amino acid changes with respect to the SARS-CoV homologs (Figure 6b,c).

### 3.6. N Protein Epitope Analysis

**Figure 7:**
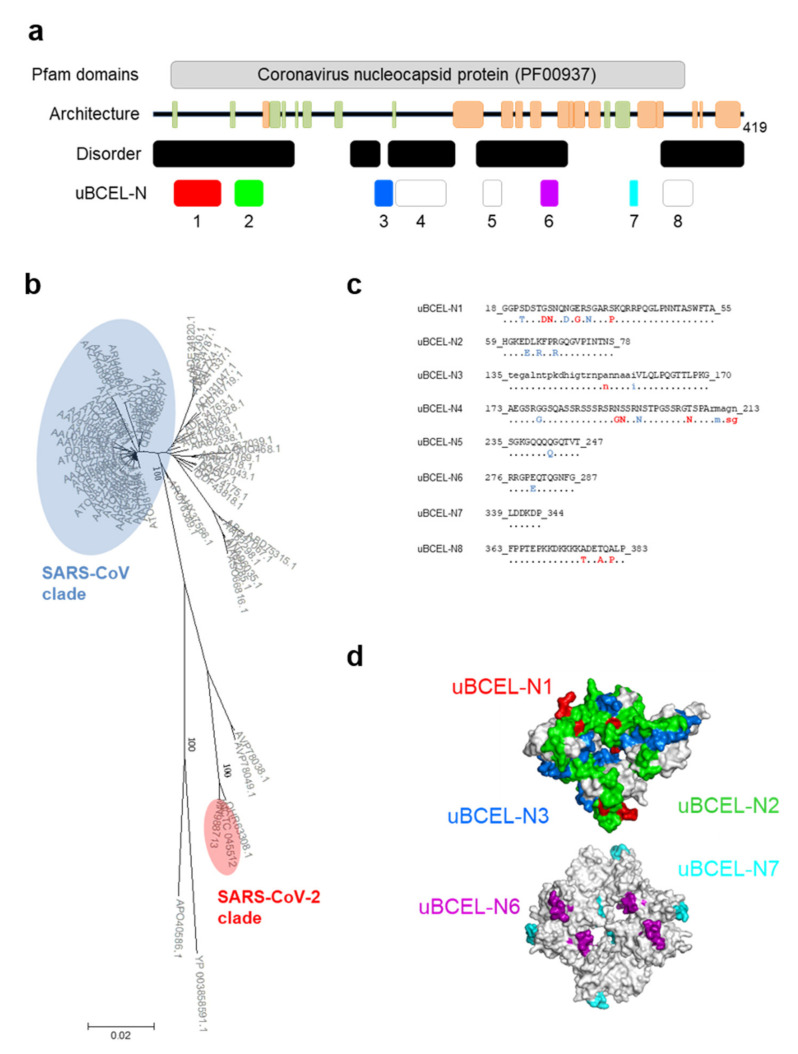
Architecture and analysis of uBCELs of nucleocapsid N protein. (**a**) a graphical depiction of N protein with Pfam domains, architecture, disorder and uBCELs localization. Color blocks: Pfam domains (grey), alpha-helices (orange), beta-strand (green). In addition, each uBCEL is shown in its own color; (**b**) phylogenetic tree of 71 non-redundant coronavirus N sequences calculated by the Neighbor-Joining method. Bootstrap values of 100 are indicated; (**c**) SARS-CoV-2 uBCEL-N sequence and changes observed in relation to SARS-CoV N protein: capital letters indicate epitopes, residues conserved ≥90% sequences (dots), changed to unique option (≥90%, red), ambiguous changes (two or more residue option in >10% sequences, blue); (**d**) structural mapping of uBCELs on the surface view of resolved three-dimensional structures for the nucleotide-binding (above) and oligomerizing (below) domains. Each uBCEL is depicted in the same colors as in Figure 7a. The uBCEL-N4, uBCEL-N5 and uBCEL-N8 are located in regions of unsolved structure and therefore are not shown.

In contrast to E and M proteins, predictions based on residue content as well as partial structural information indicate that the N protein is a remarkably disordered and antigenic polypeptide (Figure 7a). Three out of the eight uBCELs identified in this protein were ≥ 35 residues in length. Five uBCELs were highly conserved (≤1 change) compared to the SARS-CoV clade, whereas uBCEL-N1, -N4 and -N8 showed 3–5 unambiguous changes, most of them in the predicted epitope sequences (Figure 7b,c). The disordered nature of N is evident in uBCEL-N2 and -N3, which intermingled in the resolved RNA-binding domain (Figure 7d).

### 3.7. Assessment of the Agreement between uBCELs in SARS-CoV-2 and Linear B-Cell Epitopes Previously Reported for SARS-CoV

**Figure 8:**
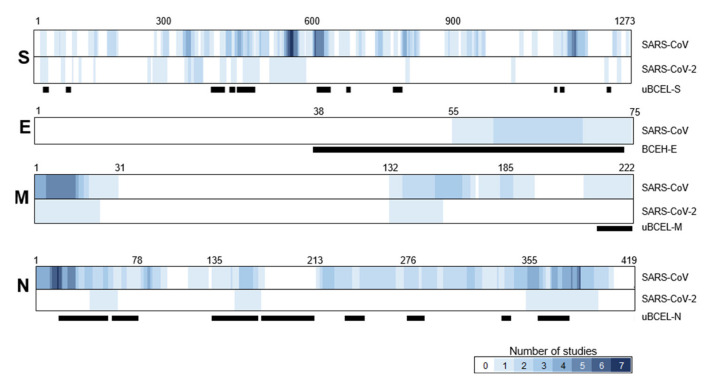
Location of previously validated linear B-cell epitopes in coronavirus virion proteins. Heatmaps indicate the bibliographic consensus of studies including this residue as part of a B-cell epitope in reported studies for SARS-CoV (*n* = 43) and SARS-CoV-2 (*n* = 7). Black bars indicate the position of uBCELs identified in this study. To the best of our knowledge, no B-cell epitopes have been reported for SARS-CoV-2 E protein. Key residues positions are indicated in each protein for identification.

Given the recent nature of the SARS-CoV-2 pandemic, the body of antigenic knowledge regarding this coronavirus is still scarce. In contrast, SARS-CoV has been analyzed over almost two decades. To determine the degree of locational novelty in the epitopes proposed in this study, they were overlaid with SARS-CoV epitopes available in immunologic and bibliographic databases (Figure 8). A total of 117 validated epitopes for SARS-CoV were identified in the four structural proteins: S (*n* = 64), E (*n* = 2), M (*n* = 11) and N (*n* = 40) (Supplementary Table S2). Twenty-four epitopes have been reported in the S1B subunit of SARS-CoV, which globally overlapped with uBCEL-S3, -S4 and -S5. Likewise, the remaining amino-terminal half of the S protein contained 39 previously validated SARS-CoV epitopes, which partially covered uBCEL-S1 and -S2, but not uBCEL-S7 from this study. In contrast, uBCEL-S1, -S2, -S8, and -S11 are found in regions with no overlap with epitopes previously described for SARS-CoV (Figure 8). The only two previously validated epitopes reported for E protein were also partially redundant with BCEH-E1. Five and six validated epitopes have been reported for the amino and carboxyl extremes of the M protein. The uBCEL-M1 presents redundancy with one of the later [33]. The N protein contained 40 previously validated epitopes, 20 in each half of the protein. The uBCEL-N1 and uBCEL-N8 are localized in highly redundant zones. Overall, the uBCELs predicted in this study show some degree of overlap with previously-validated SARS-CoV epitopes; however, redundancy is generally poor and partial, and there is no agreement with the structural criteria used in the present study.

### 3.8. Epitope Conservation in Bat Coronaviruses

Three coronavirus samples extracted from bats in China were recurrently found close to the SARS-CoV-2 clade in the phylogenetic trees generated for the four proteins. Bat-SL-CoVZXC21 (NCBI sample: MG772934.1, July 2015) and bat-SL-CoVZC45 (Sample: MG772933.1, February 2017) were isolated from *Rhinolophus sinicus* during the same study [55]. RaTG13, isolated from *Rhinolophus affinis* (Sample: MN996532.1, July 2013), is a well-known isolate that shares high global similarity to SARS-CoV-2 and is therefore considered a potential ancestor of the human lineage causing the current pandemic [50]. Thus, it is of interest to calculate the degree of conservation between the candidate uBCELs identified in this study and these sequences in order to assess the possibility of epitope switching in these bat coronaviruses. While the SARS-CoV reference sequences shared 76.9% identical residues and only six identical uBCELs compared to SARS-CoV-2, Bat-SL-CoVZXC21, and bat-SL-CoVZC45 reached 80% uBCEL residue identity and 11 identical uBCELs (Supplementary Data S1). Interestingly, RaTG13 showed 95.7% uBCEL residue identity and 14 uBCELs were a 100% match, with only a small divergence in the potential switched loops.

## 4. Discussion

In addition to discontinuous B-cell epitopes, viral linear epitopes located in protein loops have also proven useful in the biomedical field. In this study, we aimed to further exploit the linear epitope strategy by adding additional criteria to refine the selection pipeline, namely application of a consensus of epitope predictors, consideration of the antigen architecture, and the placement of each individual epitope into an evolutionary context.

A total of 20 uBCELs were identified in the four SARS-CoV-2 virion proteins using this approach. In addition, a region containing a globular minifold in the E protein was also included in our approach given the prediction of a B-cell epitope in this section and its assumed folding completeness. The most divergent uBCELs with respect to SARS-CoV were found in the S_1_ subunit. The longest uBCELs and the highest uBCEL protein coverage corresponded to the N protein, very likely because of its higher structural disorder.

The poor agreement observed between SARS-CoV-2 epitopes reported in the literature, and between previous studies and our uBCEL dataset, underscore the influence of the immunoinformatic strategy on the resulting epitope list. Differences in epitope identification selection criteria could radically affect the success of the downstream processes that employ the identified sequences.

Since the genomes of SARS-CoV and SARS-CoV-2 share 89% nucleotide identity [2], the wealth of information obtained for the former since 2003 may assist the development of antibody-based products for the current pandemic. Some recurrent, well-studied epitopes in SARS-CoV partially match with our uBCEL (see Table 1 and Supplementary Table S2). For example, in previous studies, convalescent sera with high neutralizing activity have been demonstrated to recognize epitopes with sequences related to uBCEL-S6/-S7, BCEH-E1, uBCEL-M1, and uBCEL-N1/-N3/-N8. In addition, uBCEL-S4 and -S5 are found in the S1B domain and include residues that directly interact with the ACE2 receptor. Several validated epitopes with similar sequences have been studied in SARS-CoV. Furthermore, the uBCEL-S9 and -S10 are equivalent to epitopes recognized by neutralizing antibodies that prevent viral entry into the cell [56]. Hence, antibodies against these uBCELs may explicitly neutralize the SARS-CoV-2 attachment process. Finally, we have identified five novel epitopic regions within the S protein, whose inclusion in future assays offers a chance to explore novel antigenic features in the coronavirus.

Several prophylactic strategies, each with associated strengths and limitations, have been proposed to protect humans against viral infections [47]. These include the utilization of genetically-modified attenuated viruses, purified subunits (typically proteins), and nucleic acid molecules. Although limitations remain to be addressed, DNA-based platforms have enormous potential in development of vaccines for infectious diseases [57]. For instance, epitopic sections below the folding domain length range of approximately ≤ 40 residues, can be included in DNA vaccines. Importantly, we observed that BCELs contain few and intermediate-affinity HLA-II epitopes (data not shown) and may therefore need to be combined with strong and promiscuous CD4+ T-cell epitopes that enhance their mild immunogenicity. Our approach intends to pave the way for exploiting this route by merging uBCELs from the four virion proteins in tandem and circumventing the UPR in cells transfected with the DNA vaccine. The 437 residues in our epitope dataset permit a reduction of at least 4.5-fold of the virion proteome, thus facilitating the construction of chimeric proteins including only highly antigenic sequences. Antigen engineering involving the incorporation of protein loops into chimeric proteins to improve protection has already been applied in other viruses such as papillomavirus [58] and Zika [54].

The exploration of the linear epitope option in corovaviruses is additionally warranted due to the conformational re-arrangements observed in the S protein during infection [59], which may alter the stability of discontinuous epitopes, and the extraordinarily disordered nature of the N protein. The modular nature of our approach also permits the evaluation of synergistic effects between different epitopes in animal models. In addition, it may promote the identification of non-neutralizing immunodominant epitopes that divert the immune response towards enhancement of infectivity and eosinophilia-related immunopathology by eliciting an unbalanced antiinflammatory cytokine and T1/T2 responses [60,61].

The strong sequence divergence found in five uBCELs of the S protein indicates that these are important drivers in coronavirus evolution and may play a role in the lack of immunoprotective compatibility observed between SARS-CoV and SARS-CoV-2 [62,63]. Epitope switching in loops leading to immunologically independent lineages has been observed in VIH and influenza virus [64,65]. We postulate that some of the uBCELs described here are also subjected to high selective pressure that promotes epitope switching, and that this aspect combines with glycan epitope masking and conformational changes [66] to hamper the memory response in natural animal reservoirs. Moreover, equivalent events may also take place in humans if the pandemic is prolonged over a long period of time. The incidence of residue changes in uBCELs observed in SARS-CoV-2 genomes sampled from humans to date is still low. However, the local and international dissemination of some lineages harboring up to two residue polymorphisms in uBCELs only five months after the original outbreak is of special concern. The progressive accumulation of such variants may result in immune evasion, i.e., serotypes, of the polyclonal antibody response. This would have serious consequences, such as reinfection of previously-immunized humans, vaccine escape, and false negatives in antibody-based rapid diagnostics. In this context, the existence of coronaviruses detected in bats showing intermediate epitopic features at different stages between SARS-CoV and SARS-CoV-2 is of great interest. Although virus sampling in these animals is infrequent, three bat-CoVs isolates embodied such middle links. In particular, equivalent uBCELs in RatG13—isolated as early as in July 2013—were surprisingly similar to those in SARS-CoV-2, indicating epitope migration between SARS-CoV and SARS-CoV-2 could have essentially occurred seven years ago, or earlier. Since bats are a natural reservoir for coronaviruses, epitope switch by mutation and recombination may be facilitated by the recursive attempts of coronaviruses to re-infect bats that have been previously immunized against former strains.

Our uBCEL list includes a landscape of constant and changing epitopes that are located both inside and outside of the conformational rearrangement zone, and include both glycosylated and non-glycosylated sequences in S protein. This gradient in conservation may help to elicit a balance between strong specific response against SARS-CoV-2 as well as cross-protection to future coronavirus variants. However, in the event that such cross-protection between present and as yet unidentified coronaviruses is ineffective, similar vaccines and specific antibodies for diagnostic tests and passive therapy may be promptly redesigned. Loop sequences with equivalent coordinates could be introduced into the same DNA framework immediately after the first sequenced genome of an emerging pathogenic coronavirus is available.

Although some redundancy has been observed between published epitopes and our uBCELs, our sequence combination is unique and the exact loop limits have been carefully delineated to avoid structural regions. We anticipate that the inclusion of partially structured epitopes in DNA molecules may compromise its success by inducing the UPR-related proteolysis and probably apoptosis. Such an outcome would negate the epitope integrity, presentation to naïve B-cells, and protective capacity. Our dataset follows a unified view at the service of strategies aimed at producing antibodies for different biomedical purposes. While the epitopes identified in this study will need to be experimentally tested, our characterized and rationalized catalog of epitopes is therefore of interest for vaccine developers and the general scientific community.

## 5. Conclusions

Vaccines against SARS-CoV-2 are needed to stopping deaths and the global economic drain caused by the current pandemic. Epitope-based nucleic acid vaccines, one of the most promising vaccine candidates, can trigger the unfolded protein response in host cells, thus reducing the amount of antigen available for immune stimulation and decreasing vaccine efficacy. To avoid this, all available methodologies for B-cell epitope prediction have been unified and a structural filter added in order to identify twenty loops in virion proteins enriched in B cell epitopes.

Mutational hotspots were observed in several of these regions with respect to the SARS-CoV 2003 pandemic virus. These are indicators of epitopic switch, which occurs to gradually evade the host immune response. This is strongly supported by the fact that some bat beta-coronavirus sequences are phylogenetically halfway between both human pandemics. Although unstructured epitopic zones are generally identical within SARS-CoV-2 human samples, up to two residue changes have been identified in some isolates. Exhaustive surveillance of epitopic mutations would therefore be highly recommended in order to predict future vaccine escape.

This novel epitope zone dataset is tailored to a range of prophylactic strategies that intend to elicit protective humoral responses, but could be hindered by protein misfolding. The results of this study may therefore have broad applications in complementing current initiatives aimed at developing immune-based therapeutics for SARS-CoV-2.

## Figures and Tables

**Table 1:** Epitopes identified in SARS-CoV-2 virion proteins.

Protein	uBECL or BCEH ^a^	uBCE ^b^ Location	uBCEL or BCEH Location	Flanking SS ^c^	uBCEL Sequence ^d^
S	uBCEL-S1	21–28	16–28	SP-B1	vnlttRTQLPPAY
	uBCEL-S2	71–81	68–85	B3-B4	ihvSGTNGTKRFDNpvlp
	uBCEL-S3	404–412	402–429	B25-B26	irGDEVRQIAPgqtgkiadynyklpddf
	uBCEL-S4	440–445	440–450	B26-B27	NLDSKVggnyn
	uBCEL-S5	459–470 473–480	455–491	B27-B28	lfrkSNLKPFERDISTeiYQAGSTPCngvegfncyfp
	uBCEL-S6	615–630	615–642	B38-B39	VNCTEVPVAIHADQLTptwrvystgsnv
	uBCEL-S7	676–687	676–689	B43-B44	TQTNSPRRARSVas
	uBCEL-S8	783–797	783–803	H3-B48	AQVKQIYKTPPIKDFggfnfs
	uBCEL-S9	1125–1131	1125–1131	B60-B61	NCDVVIG
	uBCEL-S10	1137–1147	1136–1147	B61-H12	TVYDPLQPELDS
	uBCEL-S11	1240–1246	1238–1246	H15p-H16p	tsCCSCLKG
E	BCEH-E1	57–68	38–75	H3p	rlcayccnivnvslvkpsfYVYSRVKNLNSSRvpdllv
M	uBCEL-M1	209–215	209–222	B10-Ct	DHSSSSDniallvq
N	uBCEL-N1	16–48	18–55	B1p-B2	GGPSDSTGSNQNGERSGARSKQRRPQGLPNNTASWFTA
	uBCEL-N2	59–78	59–78	B2-H1	HGKEDLKFPRGQGVPINTNS
	uBCEL-N3	158–170	135–170	B8-B9	tegalntpkdhigtrnpannaaiVLQLPQGTTLPKG
	uBCEL-N4	173–208	173–213	B9-H2p	AEGSRGGSQASSRSSSRSRNSSRNSTPGSSRGTSPArmagn
	uBCEL-N5	235–247	235–247	H2p-H3p	SGKGQQQQGQTVT
	uBCEL-N6	276–287	276–287	H4-H5	RRGPEQTQGNFG
	uBCEL-N7	339–344	339–344	B11-H9	LDDKDP
	uBCEL-N8	363–383	363–383	H10-H11p	FPPTEPKKDKKKKADETQALP

^a^ uBCEL: unstructured B-cell epitope loop. BCEH: B-cell epitope helix. ^b^ uBCE: unstructured B-cell epitopes. ^c^ SS: secondary structure; B: beta-strand (plus its index in the protein); H: alpha-helix (plus its index in the protein); SP: signal peptide; p: predicted by PSIPRED; Ct: C-terminus. ^d^ The uBCE zone is in capitals. The rest of the loop is in lowercase.

**Table 2:** Prevalent changes observed within uBCELs in SARS-CoV-2. Occurrence and geotemporal data is provided for residue variants found in ≥2 isolates.

uBCEL	Change(s)	*n*	Date of First Isolation	Geolocation
uBCEL-S2	I68-	11	15/03/2020	USA: WA
	N74K	2	20/01/2020	Brasil; China
	D80Y	2	31/03/2020	USA: WA
uBCEL-S5	G476S	7	10/03/2020	USA: WA
	V483A	11	05/03/2020	USA: WA
uBCEL-S7	Q677H	2	19/03/2020	USA: UT
uBCEL-S8	T791I	6	26/02/2020	Taiwan
BCEH-E1	P71L	2	19/03/2020	USA: WA
uBCEL-N2	P67S	2	17/03/2020	USA: NY; USA: WA
uBCEL-N3	A152S	2	13/03/2020	USA: UT
uBCEL-N4	S180I	2	31/03/2020	USA: WA
	S183Y	4	17/03/2020	USA
	R185C	5	15/03/2020	USA
	R185L	2	19/03/2020	USA
	S188L	3	18/03/2020	USA
	S188P	2	13/03/2020	Taiwan
	S190I	3	17/03/2020	USA: NY
	S196L	6	29/02/2020	USA
	S197L	17	26/02/2020	Greece; Spain; USA
	S202N	7	30/01/2020	China; USA
	R203K,G204R	62	27/02/2020	Czech Republic; Greece; India; Israel; Peru; Spain; Sri Lanka; Taiwan; USA
	T205I	10	29/01/2020	China; USA
	A208G	4	16/03/2020	USA: WA; USA: NY
uBCEL-N7	P344S	2	?/01/2020	Japan
uBCEL-N8	E367-	2	16/03/2020	SA: UT; USA: WA

## Supplementary Materials

The following are available online at https://www.mdpi.com/2076-393X/8/3/397/s1, Supplementary Data S1: Conservation of B-cell epitopes in S, E, M, and N proteins across coronavirus species. Supplementary Table S1: References for databases and methods utilized in this study. Supplementary Table S2: Linear B-cell epitopes identified in spike (S), envelope (E), membrane (M), and nucleocapsid (N) proteins of SARS-CoV and SARS-CoV-2 that have been validated in vivo.

## Abbreviations

List of Non-Standard Abbreviations Utilized in This Work:

BCE	B-cell epitope
BCEH	B-cell epitope in an alpha-helix section
E	envelope protein
IEDB	immune epitope database
M	membrane protein
N	nucleocapsid protein
S	spike protein
SARS-CoV	Severe Acute Respiratory Syndrome Coronavirus
SARS-CoV-2	Severe Acute Respiratory Syndrome Coronavirus 2
SDP	specificity determining position
TMH	transmembrane helix
uBCE	unstructured B-cell epitope
uBCEL	unstructured B-cell epitope-containing loop
UPR	unfolded protein response
