# Supplementary material for: Identification and Analysis of Unstructured, Linear B-Cell Epitopes in SARS-CoV-2 Virion Proteins for Vaccine Development
Source: Vaccines (Basel). 2020 Jul 20;8(3):397. doi: 10.3390/vaccines8030397 (PMC7564417; doi:10.3390/vaccines8030397)
Supplement: Supplementary file 1 [file vaccines-08-00397-s001.pdf]

Supplementary material to

Identification and analysis of unstructured, linear B-cell epitopes in SARS-CoV-2 virion proteins for vaccine development

Andrés Corral-Lugo, Mireia López-Siles, Daniel López, Michael J. McConnell and Antonio J. Martín-Galiano

Supplementary Data S1. Conservation of B-cell epitopes in S, E, M and N protein across coronavirus species. Independent sequence alignments between uBCELs/BCEH of SARS-CoV-2, and equivalent sequences in SARS-CoV and three bat coronavirus samples: bat-SL-CoVZXC21, bat-SL-CoVZC45 and RaTG13. For clarity, SARS-CoV and SARS-CoV-2 headers are in bold letters. Residues in uBCEs are in capital letters while the rest of the loop is in lowercase. Identical residues to SARS-CoV-2 sequences are highlighted in green. Deletions are indicated with dashes.

S protein

uBCEL-S1

```
SARS-CoV           20_ddvqaPNYTQHTS_32
Bat-SL-CoVZXC21    17_-dltGRTPLNPNY_28
Bat-SL-CoVZC45     17_vnltGRTPLNPNY_29
RaTG13             15_vnlttRTQLPPAY_27
SARS-CoV-2         15_vnlttRTQLPPAY_27
                        :
```

uBCEL-S2

```
SARS-CoV           72_in-------HTFGNpvip_82
Bat-SL-CoVZXC21    68_lttN-NAATKRTDNpild_84
Bat-SL-CoVZC45     69_lttN-NAATKRTDNpild_85
RaTG13             68_ihvSGTNGIKRFDNpvlp_85
SARS-CoV-2         68_ihvSGTNGTKRFDNpvlp_85
                      :        :  .**::
```

uBCEL-S3

```
SARS-CoV           389_vkGDDVRQIAPgqtgviadynyklpddf_416
Bat-SL-CoVZXC21    397_irFSEVRQVAPgqtgviadynyklpddf_424
Bat-SL-CoVZC45     398_irFSEVRQVAPgqtgviadynyklpddf_425
RaTG13             402_itGDEVRQIAPgqtgkiadynyklpddf_429
SARS-CoV-2         402_irGDEVRQIAPgqtgkiadynyklpddf_429
                       :  .:***:****** ************
```

uBCEL-S4

```
SARS-CoV           427_NIDATStgnyn_437
Bat-SL-CoVZXC21    435_KQDT-----gh_440
Bat-SL-CoVZC45     436_KQDV-----gn_441
RaTG13             440_HIDAKEggnfn_450
SARS-CoV-2         440_NLDSKVggnyn_450
                       : *       :
```

uBCEL-S5

```
SARS-CoV            442_ylrhGKLRPFERDISNVPFSPDGKPCtp-palncywp_477
Bat-SL-CoVZXC21     445_shrsTKLKPFERDLSSDENG--------------vrt_467
Bat-SL-CoVZC45      446_shrsTKLKPFERDLSSDENG--------------vrt_468
RaTG13              455_lfrkANLKPFERDISTEIYQAGSKPCngqtglncyyp_491
SARS-CoV-2          455_lfrkSNLKPFERDISTEIYQAGSTPCngvegfncyfp_491
                          *  :*:*****:*.
```

uBCEL-S6

```
SARS-CoV            601_VNCTDVSTAIHADQLTpawriystgnnv_628
Bat-SL-CoVZXC21     591_VNCTDVPTTIHADQLTpawriyaigtsv_618
Bat-SL-CoVZC45      592_VNCTDVPTTIHADQLTpawriyatgtnv_619
RaTG13              615_VNCTEVPVAIHADQLTptwrvystgsnv_642
SARS-CoV-2          615_VNCTEVPVAIHADQLTptwrvystgsnv_642
                        ****:* .:********:**:*: *..*
```

uBCEL-S7

```
SARS-CoV            662_TVSL----LRSTsq_671
Bat-SL-CoVZXC21     652_TASI----LRSTgq_661
Bat-SL-CoVZC45      653_TASI----LRSTsq_662
RaTG13              676_TQTNS----RSVas_685
SARS-CoV-2          676-TQTNSPRRARSVas-689
                        * :      **...
```

uBCEL-S8

```
SARS-CoV            765_AQVKQMYKTPTLKYFggfnfs_785
Bat-SL-CoVZXC21     755_AQVKQIYKTPPIKDFggfnfs_775
Bat-SL-CoVZC45      756_AQVKQIYKTPPIKDFggfnfs_776
RaTG13              779_AQVKQIYKTPPIKDFggfnfs_799
SARS-CoV-2          783_AQVKQIYKTPPIKDFggfnfs_803
                        *****:**** :* *******
```

uBCEL-S9

```
SARS-CoV           1107_NCDVVIG_1113
Bat-SL-CoVZXC21    1097_NCDVVIG_1103
Bat-SL-CoVZC45     1098_NCDVVIG_1104
RaTG13             1121_SCDVVIG_1127
SARS-CoV-2         1125_NCDVVIG_1131
                        .******
```

uBCEL-S10

```
SARS-CoV           1118_TVYDPLQPELDS_1129
Bat-SL-CoVZXC21    1108_TVYDPLQPELDS_1119
Bat-SL-CoVZC45     1109_TVYDPLQPELDS_1120
RaTG13             1132_TVYDPLQPELDS_1143
SARS-CoV-2         1136_TVYDPLQPELDS_1147
                        ************
```

uBCEL-S11

```
SARS-CoV           1220_tsCCSCLKG_1228
Bat-SL-CoVZXC21    1210_tsCCSCLKG_1218
Bat-SL-CoVZC45     1211_tsCCSCLKG_1219
RaTG13             1234_tsCCSCLKG_1242
SARS-CoV-2         1238_tsCCSCLKG_1246
                        *********
```

E protein

BCEH-E1

```
SARS-CoV            38_rlcayccnivnvslvkptvYVYSRVKNLNSSEgvpdllv_76
Bat-SL-CoVZXC21     38_rlcayccnivnvslvkpsfYVYSRVKNLNSSR-vpdllv_75
Bat-SL-CoVZC45      38_rlcayccnivnvslvkpsfYVYSRVKNLNSSR-vpdllv_75
RaTG13              38_rlcayccnivnvslvkpsfYVYSRVKNLNSSR-vpdllv_75
SARS-CoV-2          38_rlcayccnivnvslvkpsfYVYSRVKNLNSSR-vpdllv_75
                       *****************:.************. ******
```

M protein

uBCEL-M1

```
SARS-CoV            208_DHAGSNDniallvq_221
Bat-SL-CoVZC45      209_DHSSSSDniallvq_222
Bat-SL-CoVZXC21     209_DHSSSSDniallvq_222
RaTG13              209_DHSSSSDniallvq_222
SARS-CoV-2          209_DHSSSSDniallvq_222
                        **:.*.********
```

N protein

uBCEL-N1

```
SARS-CoV           19_GGPTDSTDNNQNGGRNGARPKQRRPQGLPNntaswfta_56
Bat-SL-CoVZXC21    18_GGPSDSSDNSQNGERNGARPKQRRPQGLPNntaswfta_55
Bat-SL-CoVZC45     18_GGPSDSSDNSKNGERNGARPKQRRPQGLPNntaswfta_55
RaTG13             18_GGPSDSTGSNQNGERSGARPKQRRPQGLPNntaswfta_55
SARS-CoV-2         18_GGPSDSTGSNQNGERSGARSKQRRPQGLPNntaswfta_55
                      ***:**:...:** *.*** ******************
```

uBCEL-N2

```
SARS-CoV           60_HGKEELRFPRGQGVPINTNS_79
Bat-SL-CoVZXC21    59_HGKENLTFPRGQGVPINTNS_78
Bat-SL-CoVZC45     59_HGKENLTFPRGQGVPINTNS_78
RaTG13             59_HGKEDLKFPRGQGVPINTNS_78
SARS-CoV-2         59_HGKEDLKFPRGQGVPINTNS_78
                      ****:* *************
```

uBCEL-N3

```
SARS-CoV          136_tegalntpkdhigtrnpnnnaatVLQLPQGTTLPKG_171
Bat-SL-CoVZXC21   135_tegalntpkdhigtrnpannaaiVLQLPQGTTLPKG_170
Bat-SL-CoVZC45    135_tegalntpkdhigtrnpannaaiVLQLPQGTTLPKG_170
RaTG13            135_tegalntpkdhigtrnpannaaiVLQLPQGTTLPKG_170
SARS-CoV-2        135_tegalntpkdhigtrnpannaaiVLQLPQGTTLPKG_170
                      ***************** **** *************
```

uBCEL-N4

```
SARS-CoV          174_AEGSRGGSQASSRSSSRSRGNSRNSTPGSSRGNSPArmasg_214
Bat-SL-CoVZXC21   173_AEGSRGGSQASSRSSSRSRNSSRNSTPGSSRGTSPArmagn_213
Bat-SL-CoVZC45    173_AEGSRGGSQASSRSSSRSRNSSRNSTPGSSRGTSPArmagn_213
RaTG13            173_AEGSRGGSQASSRSSSRSRNSSRNSTPGSSRGTSPArmagn_213
SARS-CoV-2        173_AEGSRGGSQASSRSSSRSRNSSRNSTPGSSRGTSPArmagn_213
                      *******************..***********.******..
```

uBCEL-N5

```
SARS-CoV          236_SGKGQQQQGQTVT_248
Bat-SL-CoVZXC21   235_SGKGQQQQGQTVT_247
Bat-SL-CoVZC45    235_SGKGQQQQGQTVT_247
RaTG13            235_SGKGQQQQSQTVT_247
SARS-CoV-2        235_SGKGQQQQGQTVT_247
                      ********.****
```

uBCEL-N6

```
SARS-CoV            277_RRGPEQTQGNFG_288
Bat-SL-CoVZXC21     276_RRGPEQTQGNFG_287
Bat-SL-CoVZC45      276_RRGPEQTQGNFG_287
RaTG13              276_RRGPEQTQGNFG_287
SARS-CoV-2          276_RRGPEQTQGNFG_287
                        ************
```

uBCEL-N7

```
SARS-CoV            340_LDDKDP_345
Bat-SL-CoVZXC21     339_LDDKDP_344
Bat-SL-CoVZC45      339_LDDKDP_344
RaTG13              339_LDDKDP_344
SARS-CoV-2          339_LDDKDP_344
                        ******
```

uBCEL-N8

```
SARS-CoV            363_FPPTEPKKDKKKKTDEAQPLP_383
Bat-SL-CoVZXC21     363_FPPTEPKKDKKKKADELQALP_383
Bat-SL-CoVZC45      363_FPPTEPKKDKKKKADELQALP_383
RaTG13              363_FPPTEPKKDKKKKADETQALP_383
SARS-CoV-2          363_FPPTEPKKDKKKKADETQALP_383
                        *************:** * **
```

Supplementary Table S1. References for databases and methods utilized in this study.

Method/database	Application	Reference
AAPPred	Prediction of linear B-cell epitopes	[1]
ABCPred	Prediction of linear B-cell epitopes	[2]
Bepipred	Prediction of linear B-cell epitopes	[3]
Bepipred 2.0	Prediction of linear B-cell epitopes	[4]
BLAST	Sequence database searching	[5]
CD-HIT 4.8.1	Sequence clustering	[6]
Clustal Omega 1.2.4	Sequence alignment	[7]
COILS	Prediction of coiled-coil regions	[8]
DISOPRED 3.1	Prediction of disordered regions	[9]
Emini	Prediction of accessibility	[10]
IUPRED2	Prediction of unstructured regions	[11]
Janin	Prediction of exposed surface	[12]
Karplus	Prediction of flexibility	[13]
Kolaskar	Prediction of antigenicity	[14]
LBEEP	Prediction of linear B-cell epitopes	[15]

Mega 7	Environment for phylogeny analysis	[16]
NCBI	Sequence database for searching	[17]
Neighbor-Joining	Calculation of phylogeny	[18]
NetMHCpan EL 4.0	Prediction of HLA-1 epitopes	[19]
NetMHCIIpan 3.2	Prediction of HLA-2 epitopes	[20]
Parker	Prediction of hydrophilicity	[21]
Pellenquer	Prediction of turns	[22]
Pfam 32.0	Identification of protein domains	[23]
Phobius	Prediction of transmembrane helices	[24]
Poison correction	Calculation of phylogenetic branch lengths	[25]
Ponnuswamy	Prediction of polarity	[26,27]
PSI-PRED 4.0	Prediction of secondary structure	[27]
SDPPred	Identification of SDPs	[28]
SignalP 5.0	Prediction of signal peptide	[29]
SVMtrip	Prediction of linear B-cell epitopes	[30]

Supplementary Table S2. Linear B-cell epitopes identified in spike (S), envelope (E), membrane (M), and nucleocapsid (N) proteins of SARS-CoV and SARS-CoV-2 that have been validated *in vivo*.

Structural proteins and subunits			Epitope		Coronavirus	Experiment type	Experimental evidence	Reference
			**Location (residues)**	**Sequence**				
S	S1	S1B	357-365	RISNCVADY	SARS-CoV-2	*In silico*	Not tested	[31]
			361-369	CVADYSVLY				
			369-391	YNSASFSTFKCYGVSPTK LNDLCF	SARS-CoV-2	*In vitro*	Not tested	[32]
			424-437	KLPDDFTGCVIAWN	SARS-CoV-2	*In silico*	Not tested	[33]
			447-458	NYNYLYRLFRK				
			92-96	ASTEK	SARS-CoV-2	*In silico*	Not tested	[34]
			110-115	LDSKTQ				
			352-357	AWNRKR				
			373-390	SFSTFKCYGVSPTKLNDL	SARS-CoV-2	*In silico*	Not tested	[35]
			434-467	GNYNYKYRYLRHGKLRP FERDISNVPFSPDGKPC	SARS-CoV	*In vitro*	Sera contain neutralizing IgG antibody against peptides	[36]
			460-476	FSPDGKPCTPPALNCYW	SARS-CoV	*In vitro*	Monoclonal antibodies which neutralize the virus *in vitro*	[37]
			337-360	VYAWERKKISNCVADYS VLYNSTF	SARS-CoV	*In vitro*	Construction of mimotopes that reveal key amino acid residues involved in antibody binding	[38]
			380-399	SNVYADSFVVKGDDVRQ IAP				
			491-510	YQPYRVVVLSFELLNAPA TV	SARS-CoV	*In vitro*	Epitope recognized by monoclonal antibodies with virus neutralizing activity	[39]
			437-459	NYKYRYLRHGKLRPFER DISNVP	SARS-CoV	*In silico* *in vitro* *in vivo*	Induction of cellular and humoral responses. Epitope recognized by polyclonal sera	[40]

			336-352	SVYAWERKKISNCVADY	SARS-CoV	*In silico*	No tested	[41]
			424-435	NTRNIDATSTGN				
			442-458	YLKHGKLRPFERDISNV				
			459-470	PFSPDGKPCTPP				
			483-494	FYTTTGIGYQPY				
			424-435	NTRNIDATSTGN	SARS-CoV	*In silico*<br>*in vitro*<br>*in vivo*	Peptide recognized by antibodies in sera	[42]
			536-552	GVLTPSSKRFQPFQQFG	SARS-CoV	*In vitro*<br>*in vivo*	Immunized mice produce antibodies with neutralizing activity	[43]
			335-352	SVYAWERKKISNCVADY	SARS-CoV	*In vitro*	Neutralizing antibodies that recognized epitopes in the RBD	[44]
			442-458	YLRHGKLRPFERDISNV				
			430–454	ATSTGNYNYKYRYLRHGKLRPFERD	SARS-CoV	*In silico*<br>*in vitro*	Antigen recognized by monoclonal antibody	[45]
			447-458	LRPFERDISNV	SARS-CoV	*In silico*<br>*in vitro*	Predicted epitope recognized by monoclonal antibody *in vitro*	[46]
			343-367	KKISNCVADYSVLYNSTFFSTFKCY	SARS-CoV	*In vitro*<br>*in vivo*	Antigen recognized by antibodies with virus neutralizing activity	[47]
			373-390	KLNDLCFSNVYADSFVVK				
			411-428	KLPDDFMGCVLAWNTRNI				
			323-334	CPFGEVFNATKF	SARS-CoV	*In vitro*	Convalescent sera with high neutralizing activity against SARS-CoV recognized epitopes in the peptides	[48]
			467-480	CTPPALNCYWPLND				
			471-503	ALNCYWPLNDYGFYTTTGIGYQPYRVVVLSFEL	SARS-CoV-2	*In silico*<br>*in vitro*	Antibodies against a peptide located in the receptor binding domain block binding and entrance of SARS-CoV into host cells in vitro	[49]
			287-317	DAVDCALDPLSETKCTLKSFTVEKGIYQTSN	SARS-CoV-2	*In silico*	Not tested	[50]

			524-598	VCGPKKSTNLVKNKCVNFNFNGLTGTGVLTESNKKFLPFQQFGRDIADTTDAVRDPQTLEILDITPCSFGGVSVI				
			815-823	RSFIEDLLF	SARS-CoV-2	*In silico*	Not tested	[31]
			1039-1047	RVDFCGKGY				
			1237-1245	MTSCCSCLK				
			1264-1272	VLKGVKLHY				
			144-153	YYHKNNKSWM	SARS-CoV-2	*In silico*	Not tested	[34]
			277-283	KYNENGT				
			15-30	CVNLTTRTQLPPAYTN	SARS-CoV-2	*In silico*	Not tested	[35]
			61-76	NVTWFHAIHVSGTNGT				
			175-201	KSGNFKHLREFVFKNKDGFLYVYKGY	SARS-CoV	*In vitro*	Sera contain neutralizing IgG against peptides	[36]
			174-195	EKSGNFKHLREFVFKNKDGFLY	SARS-CoV	*In silico*<br>*in vitro*	Induction of cellular and humoral responses. Epitope recognized by polyclonal sera	[40]
			91-102	ATEKSNVVRGWV				
			291-308	KSFEIDKGIYQTSNFRVV	SARS-CoV	*In vitro*<br>*in vivo*	Inoculation induces IgG and IgA in mice that exhibit potent neutralizing activity against SARS-CoV	[51]
			130-150	FELCDNPFFAVSKPMGTQTHT	SARS-CoV	*In vitro*	Antibodies block the binding of S protein to Vero E6 cells	[52]
			49–62	SDTLYLTQDLFLPF	SARS-CoV	*In vitro*	Convalescent sera with high neutralizing activity against SARS-CoV recognized epitopes in the peptides	[48]
			295–306	IDKGIYQTSNFR				
	**S**		601-640	GTNTSNQVAVLYQDVNCTEVPVAIHADQLTPTWRVYSTGS	SARS-CoV-2	*In silico*	Not tested	[21]
			802-819	FSQILPDPSKPSKRSFIE				

		888-909	FGAG LQIPFAMQMAYRFNG				
	2	814-826	KRSFIEDLLFNKV	SARS-CoV-2	*In silico*	Not tested	[53]
		560-571	LPFQQFGRDIAD	SARS-CoV-2	*In silico*	Not tested	[33]
		754-764	LQYGSFCTQLN				
		789-799	YKTPPIKDFGG				
		1139-1152	DPLQPELDSFKEEL				
		527-530	PKKS	SARS-CoV-2	*In silico*	Not tested	[34]
		601-606	GTNTSN				
		689-700	YQTQTNSPRRAR				
		771-779	EQDKNTQ				
		1154-1162	KYFKNHTSP				
		1141-1184	LQPELDSFKEELDKYFKN HTSPDVDLGDISGINASV VNIQKEID	SARS-CoV-2	*In vitro*	Neutralizing antibodies recognize the S2 domain of S protein and confers protection against a wide range of SARS-CoVs	[54]
		1128-1159	DSFKEELDKYFKNHTSPD VDLGDISGINASVV				
		958-966	VLNDILSRL	SARS-CoV	*In vitro*	Sera contained neutralizing IgG antibody against peptides	[36]
		1156−1178	ASVVNIQKEIDRLNEVAK NLNES	SARS-CoV	*In silico* *in vitro* *in vivo*	Sera from vaccinated mice contain IgG that exhibit virus neutralization activity	[55]
		556-568	SDFTDSVRDPKTS	SARS-CoV	*In silico* *in vitro*	Induction of cellular and humoral responses. Epitope recognized by polyclonal sera	[40]
		915-949	QIQESLTTTSTALGKLQD VVNQNAQALNTLVKQLS	SARS-CoV	*In silico*	Not tested	[41]
		1150-1184	ISGINASVVNIQKEIDRLN EVAKNLNESLIDLQE				
		1065-1076	HEGKAYFPREGV	SARS-CoV	*In silico* *in vitro* *in vivo*	Peptide recognized by antibodies in sera	[56]
		520-537	IKNQCVNFNFNGLTGTGV	SARS-CoV	*In vitro*	Inoculation in mice induces IgG	[51]

		564-581	DPKTSEILDISPCSFGGV		*in vivo*	and IgA that exhibit a potent neutralizing activity against SARS-CoV	
		540-554	PSSKRFQPFQQFGRD	SARS-CoV	*In silico* *in vitro*	Peptides at the N- the C-terminal regions of the N protein elicited strong antibody responses.	[57]
		553-570	RDVSDFTDSVRDPKTSEI				
		1236-1255	CKFDEDDSEPVLKGVKL HYT				
		1091-	NFFSPQIITTDNTFVSGNC DVVIGIINNTVYDPLQPEL DSFKEELDKYFKNHTSPD VDLGDISGINASVVNIQK EIDRLNEVAKNLNESLID LQELGKYEQYI	SARS-CoV	*In vitro*	Antibodies recognized HR2 domain and neutralize virus infection *in vitro*	[58]
		1143-1157	SPDVDLGDISGINAS	SARS-CoV	*In vitro*	Antibodies recognized HR2 domain	[59]
		539-559	TPSSKRFQPFQQFGRDVS DFT	SARS-CoV	*In vitro*	Four epitopes in S1 domain induce a humoral response in mice	[60]
		548-567	FQQFGRDVSDFTDSVRDP KT				
		583-606	VITPGTNASSEVAVLYQD VNCTDV				
		607-630	STAIHADQLTPAWRIYST GNNVFQ				
		927–937	GLGKLQDVVNQNGE	SARS-CoV	*In vitro*	Antibodies recognized SARS-CoV	[61]
		942–951	ALNTLVKQLSSNC				
		789-799	PDPLKPTKRSF	SARS-CoV	*In silico* *in vitro*	Epitope recognized by monoclonal antibody	[46]
		540-559	PSSKRFQPFQQFGRDVSD FT	SARS-CoV	*In silico* *in vitro* *in vivo*	Antibodies without neutralizing activity but inhibit syncytia formation	[62]
		731–753	CANLLLQYGSFCTQLNR ALSGIA				
		786-811	QILPDPLKPTKRSFIEDLL FNKVTLA	SARS-CoV	*In vitro*	Convalescent sera recognize S2 domain	[63]
		490-510	GYQPYRVVVLSFELLNAP	SARS-CoV	*In vitro*	Antibodies block the binding of S	[52]

			AT			protein to Vero E6 cells	
		599-620	QDVNCTDVSTAIHADQL TPAWR	SARS-CoV	*In silico* *in vitro*	Sera from patients with SARS-CoV react with peptides	[64]
		947-965	QLSSNFGAISSVLNDILSR				
		607-627	STAIHADQLTPAWRIYST GN	SARS-CoV	*In vitro*	Peptide recognized by monoclonal antibodies which neutralize SARS-CoV	[65]
		599-620	QDVNCTDVSTAIHADQL TPAWR	SARS-CoV	*In silico* *in vitro*	Peptide recognized by antibodies in sera	[66]
		597-603	LYQDVNC	SARS-CoV	*In vitro*	Immunization with this peptide induces antibodies that enhance infection both *in vitro* and in non-human primate	[67]
		731-745	CANLLLQYGSFCTQL	SARS-CoV	*In vitro* *in vivo*	Antibodies recognize the cleavage site critical for SARS-CoV entry into host cells	[68]
		545-558	FQPFQQFGRDVSDF	SARS-CoV	*In vitro*	Convalescent sera with high neutralizing activity against SARS-CoV recognized epitopes in the peptides	[48]
		553-564	RDVSDFTDSVRD				
		651-662	PIGAGICASYHT				
		663-674	VSLLRSTSQKSI				
		695–708	AIPTNFSISITTEV				
		737–748	QYGSFCTQLNRA				
		879–890	PFAMQMAYRFNG				
		791-805	PLKPTKRSFIEDLLF	SARS-CoV	*In vitro*	Peptides recognized by convalescent sera	[69]
		1111-1130	VIGVINNTVYDPLQPELD SF	SARS-CoV	*In vitro*	Peptide recognized by neutralizing antibody that prevents viral	[70]
		60-76	SRVKNLNSSEGVPDLLV	SARS-CoV	*In silico* *in vitro*	Peptides elicit antibody responses	[57]

**E**	55-70	TVYVYSRVKNLNSSEG	SARS-CoV	*In vitro*	Convalescent sera with high neutralizing activity against SARS-CoV recognized epitopes in the peptides	[48]
**M**	1-24	MADSNGTITVEELKKLLE QWNLVI	SARS-CoV-2	*In silico*	No tested	[50]
	132-151	PLLESELVIGAVILRGHLR I				
	1-31	MADNGTITVEELKQLLEQ WNLVIGFLFLAWI	SARS-CoV	*In silico* *in vitro*	Peptides react with convalescent sera and induce high antibody titers in rabbits	[44]
	132-161	LMESELVIGAVIIRGHLR MAGHSLGRCDIK				
	1-20	MADNGTITVEELKQLLEQ WN	SARS-CoV	*In silico* *in vitro*	Peptide recognized by monoclonal antibody	[40]
	149-163	RMAGHSLGRCDIKDLP	SARS-CoV	*In silico* *in vitro* *in vivo*	Peptide recognized by monoclonal antibody	[42]
	173-185	PKEITVATSRTLS				
	204-221	KLNTDHAGSNDNIALLV Q				
	1-18	MADNGTITVEELKQLLEQ	SARS-CoV	*In silico* *in vitro*	Peptides elicit an antibody response	[57]
	1-15	MADNGTITVEELKQL	SARS-CoV	*In vitro*	Recognition by convalescent sera	[71]
	165–176	KEITVATSRTLS	SARS-CoV	*In vitro*	Convalescent sera with high neutralizing activity against SARS-CoV recognized epitopes in the peptides	[48]
	5–16	GTITVEELKQLL				
	137–158	LVIGAVIIRGHLRMAGHS LGR	SARS-CoV	*In silico* *in vitro*	Peptide recognized by antibodies in sera	[66]
**N**	42-62	RPQGLPNNTASWFTALT QHGK	SARS-CoV-2	*In silico*	Not tested	[50]
	153-172	NNN TVLQLPQGTTLPKGF				
	355-401	NKHIDAYKTFPPTEPKKD KKKKTDEAQPLPQRQKK QPTVTLLP DM				

	111-125	FYYLGTGPEASLPYG	SARS-CoV	*In vitro*	Peptide reacts with sera form mouse, rat, rabbit, civet, pig and horse	[72]
	1–17	MSDNGPQSNQRSAPRIT	SARS-CoV	*In silico* *in vitro*	Peptides elicit antibody responses	[57]
	13–30	APRITFGGPTDSTDNNQN				
	25–43	TDNNQNGGRNGARPKQRRP				
	38–55	PKQRRPQGLPNNIASWFT				
	341–360	DDKDPQFKDNVILLNKHIDA				
	356–375	KHIDAYKTFPPTEPKKDKKK				
	371–390	KDKKKKTDEAQPLPQRQKKQ				
	11-19	RSAPRITF	SARS-CoV	*In vitro*	Antibody binding in phage library screen	[73]
	17-32	TFGGPTDSTDNNQNGG	SARS-CoV	*In vitro*	Peptides recognized by antibodies	[74]
	135-150	ATEGALNTPKDHIGTR				
	117-132	GPEASLPYGANKEGIV				
	215-239	GGETALALLLLDRLNQLESKVSGKG				
	245-268	QTVTKKS EASKKPRQKRTATKQ				
	274-283	AFGRRGPEQT	SARS-CoV	*In vitro*	Monoclonal antibodies recognized epitopes	[75]
	286-295	NFGDQDLIRQ				
	316-325	FGMSRIGMEV				
	361-367	YKTFPPT				
	1-30	MSDNGPQSNQRSAPRITF GGPTDSTDNNQN	SARS-CoV	*In vitro*	Peptide recognition by antibodies	[76]
	81-95	PDDQIGYYRRATRRV				
	86-100	GYYRRATRRVRGGDG				
	156-175	TVLQLPQGTTLPK				
	300-320	HWPQIAQFAPSASAFFGM				

		SR				
	336-350	GAIKLDDKDPQFKDN				
	351-365	VILLNKHIDAYKTFP				
	1-69	MSDNGPQSNQRSAPRITFGGPTDSTDNNQNGGRNGARPKQRRPQGLPNNIASWFTALTQHGKEELRFPR	SARS-CoV	*In vitro*	Recognition by monoclonal antibodies	[77]
	1-20	MSDNGPQSNQRSAPRITFGG	SARS-CoV	*In vitro*	Peptides react with convalescent sera. Antibody production after peptide immunization	[78]
	150-170	RNPNNNTVLQLPQGTTLPK				
	390-410	QPTVTLLPDMDDFSRQLQN				
	371-390	KDKKKKTDEAQPLPQRQKKQ	SARS-CoV		Peptides react with convalescent sera	[79]
	385-407	QRQKKQPTVTLLP DMDDFSRQ				
	52-59	SWFTALTQ	SARS-CoV	*In silico in vitro*	Synthetic peptides react with convalescent sera and antisera from mice immunized with inactivate SARS-CoV	[80]
	83-89	DQIGYYR				
	156-166	TVLQLPQGT				
	218-227	TALALLLLDR				
	347-363	FKDNVILLNKHIDAYKT				
	379-385	EAQPLPQ				
	389-398	KQPTVTLLPA				
	66-87	QLPQGTTLPKGFYAEGSRGGSQ	SARS-CoV	*In silico in vitro*	Peptide recognized by antibodies in sera	[66]
	371–390	KDKKKKTDEAQPLPQRQKKQ				

Abbreviations:

[a] IEDB: Immune Epitope Database

[b] PISA: Proteins, Interfaces, Structures and Assemblies

[c] ViPR: Virus Pathogen Database and Analysis Resource

REFERENCES

1. Davydov Ia, I.; Tonevitskii, A.G. Linear B-cell epitope prediction. *Mol Biol* **2009**, *43*, 166-174.
2. Saha, S.; Raghava, G.P. Prediction of continuous B-cell epitopes in an antigen using recurrent neural network. *Proteins* **2006**, *65*, 40-48.
3. Larsen, J.E.; Lund, O.; Nielsen, M. Improved method for predicting linear B-cell epitopes. *Immunome Res* **2006**, *2*, 1745-7580.
4. Jespersen, M.C.; Peters, B.; Nielsen, M.; Marcatili, P. BepiPred-2.0: improving sequence-based B-cell epitope prediction using conformational epitopes. *Nucleic Acids Res* **2017**, *45*, W24-W29.
5. Altschul, S.F.; Gish, W.; Miller, W.; Myers, E.W.; Lipman, D.J. Basic local alignment search tool. *J Mol Biol* **1990**, *215*, 403-410.
6. Fu, L.; Niu, B.; Zhu, Z.; Wu, S.; Li, W. CD-HIT: accelerated for clustering the next-generation sequencing data. *Bioinformatics* **2012**, *28*, 3150-3152.
7. Sievers, F.; Higgins, D.G. Clustal Omega for making accurate alignments of many protein sequences. *Protein Sci* **2018**, *27*, 135-145.
8. Lupas, A.; Van Dyke, M.; Stock, J. Predicting coiled coils from protein sequences. *Science* **1991**, *252*, 1162-1164.
9. Jones, D.T.; Ward, J.J. Prediction of disordered regions in proteins from position specific score matrices. *Proteins* **2003**, *6*, 573-578.
10. Emini, E.A.; Hughes, J.V.; Perlow, D.S.; Boger, J. Induction of hepatitis A virus-neutralizing antibody by a virus-specific synthetic peptide. *J Virol* **1985**, *55*, 836-839.
11. Meszaros, B.; Erdos, G.; Dosztanyi, Z. IUPred2A: context-dependent prediction of protein disorder as a function of redox state and protein binding. *Nucleic Acids Res* **2018**, *46*, W329-W337.
12. Janin, J.; Wodak, S. Conformation of amino acid side-chains in proteins. *J Mol Biol* **1978**, *125*, 357-386.
13. Karplus, P.A.; Schulz, G.E. Prediction of chain flexibility in proteins - A tool for the selection of peptide antigens. *Naturwissenschaften* **1985**, *72*, 212-213, doi:10.1007/bf01195768.
14. Kolaskar, A.S.; Tongaonkar, P.C. A semi-empirical method for prediction of antigenic determinants on protein antigens. *FEBS Lett* **1990**, *276*, 172-174.
15. Saravanan, V.; Gautham, N. Harnessing Computational Biology for Exact Linear B-Cell Epitope Prediction: A Novel Amino Acid Composition-Based Feature Descriptor. *Omics* **2015**, *19*, 648-658.
16. Kumar, S.; Stecher, G.; Tamura, K. MEGA7: Molecular Evolutionary Genetics Analysis Version 7.0 for Bigger Datasets. *Mol Biol Evol* **2016**, *33*, 1870-1874.
17. Database resources of the National Center for Biotechnology Information. *Nucleic Acids Res* **2018**, *46*, D8-D13.
18. Saitou, N.; Nei, M. The neighbor-joining method: a new method for reconstructing phylogenetic trees. *Mol Biol Evol* **1987**, *4*, 406-425.
19. Hoof, I.; Peters, B.; Sidney, J.; Pedersen, L.E.; Sette, A.; Lund, O.; Buus, S.; Nielsen, M. NetMHCpan, a method for MHC class I binding prediction beyond humans. *Immunogenetics* **2009**, *61*, 1-13, doi:10.1007/s00251-008-0341-z.

20. Jensen, K.K.; Andreatta, M.; Marcatili, P.; Buus, S.; Greenbaum, J.A.; Yan, Z.; Sette, A.; Peters, B.; Nielsen, M. Improved methods for predicting peptide binding affinity to MHC class II molecules. *Immunology* **2018**, *154*, 394-406, doi:10.1111/imm.12889.
21. Parker, J.M.; Guo, D.; Hodges, R.S. New hydrophilicity scale derived from high-performance liquid chromatography peptide retention data: correlation of predicted surface residues with antigenicity and X-ray-derived accessible sites. *Biochemistry* **1986**, *25*, 5425-5432.
22. Pellequer, J.L.; Westhof, E.; Van Regenmortel, M.H. Correlation between the location of antigenic sites and the prediction of turns in proteins. *Immunol Lett* **1993**, *36*, 83-99.
23. El-Gebali, S.; Mistry, J.; Bateman, A.; Eddy, S.R.; Luciani, A.; Potter, S.C.; Qureshi, M.; Richardson, L.J.; Salazar, G.A.; Smart, A., et al. The Pfam protein families database in 2019. *Nucleic Acids Res* **2019**, *47*, D427-D432.
24. Kall, L.; Krogh, A.; Sonnhammer, E.L. A combined transmembrane topology and signal peptide prediction method. *J Mol Biol* **2004**, *338*, 1027-1036.
25. Zuckerkandl, E.; Pauling, L. Molecules as documents of evolutionary history. *J Theor Biol* **1965**, *8*, 357-366.
26. Ponnuswamy, P.K.; Prabhakaran, M.; Manavalan, P. Hydrophobic packing and spatial arrangement of amino acid residues in globular proteins. *Biochim Biophys Acta* **1980**, *623*, 301-316.
27. Buchan, D.W.A.; Jones, D.T. The PSIPRED Protein Analysis Workbench: 20 years on. *Nucleic Acids Res* **2019**, *47*, W402-W407.
28. Kalinina, O.V.; Mironov, A.A.; Gelfand, M.S.; Rakhmaninova, A.B. Automated selection of positions determining functional specificity of proteins by comparative analysis of orthologous groups in protein families. *Protein Sci* **2004**, *13*, 443-456.
29. Almagro Armenteros, J.J.; Tsirigos, K.D.; Sonderby, C.K.; Petersen, T.N.; Winther, O.; Brunak, S.; von Heijne, G.; Nielsen, H. SignalP 5.0 improves signal peptide predictions using deep neural networks. *Nat Biotechnol* **2019**, *37*, 420-423.
30. Yao, B.; Zhang, L.; Liang, S.; Zhang, C. SVMTriP: a method to predict antigenic epitopes using support vector machine to integrate tri-peptide similarity and propensity. *PLoS One* **2012**, *7*, 12.
31. Kumar, S.; Maurya, V.K.; Prasad, A.K.; Bhatt, M.L.B.; Saxena, S.K. Structural, glycosylation and antigenic variation between 2019 novel coronavirus (2019-nCoV) and SARS coronavirus (SARS-CoV). *Virusdisease* **2020**, *31*, 13-21.
32. Yuan, M.; Wu, N.C.; Zhu, X.; Lee, C.D.; So, R.T.Y.; Lv, H.; Mok, C.K.P.; Wilson, I.A. A highly conserved cryptic epitope in the receptor-binding domains of SARS-CoV-2 and SARS-CoV. *Science* **2020**, *3*.
33. Tilocca, B.; Soggiu, A.; Musella, V.; Britti, D.; Sanguinetti, M.; Urbani, A.; Roncada, P. Molecular basis of COVID-19 relationships in different species: a one health perspective. *Microbes Infect* **2020**, *17*, 30048-30044.
34. Zheng, M.; Song, L. Novel antibody epitopes dominate the antigenicity of spike glycoprotein in SARS-CoV-2 compared to SARS-CoV. *Cell Mol Immunol* **2020**, *4*, 020-0385.
35. Baruah, V.; Bose, S. Immunoinformatics-aided identification of T cell and B cell epitopes in the surface glycoprotein of 2019-nCoV. *J Med Virol* **2020**, *92*, 495-500, doi:10.1002/jmv.25698.

36. Lien, S.P.; Shih, Y.P.; Chen, H.W.; Tsai, J.P.; Leng, C.H.; Lin, M.H.; Lin, L.H.; Liu, H.Y.; Chou, A.H.; Chang, Y.W., et al. Identification of synthetic vaccine candidates against SARS CoV infection. *Biochem Biophys Res Commun* **2007**, *358*, 716-721, doi:10.1016/j.bbrc.2007.04.164.
37. Berry, J.D.; Hay, K.; Rini, J.M.; Yu, M.; Wang, L.; Plummer, F.A.; Corbett, C.R.; Andonov, A. Neutralizing epitopes of the SARS-CoV S-protein cluster independent of repertoire, antigen structure or mAb technology. *MAbs* **2010**, *2*, 53-66.
38. Zhang, X.P.; Wang, J.X.; Wen, K.; Mou, Z.R.; Zou, L.Y.; Che, X.Y.; Ni, B.; Wu, Y.Z. Antibody Binding Site Mapping of SARS-CoV Spike Protein Receptor-Binding Domain by a Combination of Yeast Surface Display and Phage Peptide Library Screening. *Viral Immunol* **2009**, *22*, 407-415, doi:10.1089/vim.2009.0046.
39. Zhong, L.; Haynes, L.; Struble, E.B.; Tamin, A.; Virata-Theimer, M.L.; Zhang, P. Antibody-mediated synergy and interference in the neutralization of SARS-CoV at an epitope cluster on the spike protein. *Biochem Biophys Res Commun* **2009**, *390*, 1056-1060, doi:10.1016/j.bbrc.2009.10.115.
40. Wang, X.H.; Xu, W.; Tong, D.Y.; Ni, J.; Gao, H.F.; Wang, Y.; Chu, Y.W.; Li, P.P.; Yang, X.M.; Xiong, S.D. A chimeric multi-epitope DNA vaccine elicited specific antibody response against severe acute respiratory syndrome-associated coronavirus which attenuated the virulence of SARS-CoV in vitro. *Immunol Lett* **2008**, *119*, 71-77, doi:10.1016/j.imlet.2008.04.005.
41. El-Manzalawy, Y.; Dobbs, D.; Honavar, V. Predicting linear B-cell epitopes using string kernels. *J Mol Recognit* **2008**, *21*, 243-255, doi:10.1002/jmr.893.
42. Yu, H.; Jiang, L.F.; Fang, D.Y.; Yan, H.J.; Zhou, J.J.; Zhou, J.M.; Liang, Y.; Gao, Y.; Zhao, W.; Long, B.G. Selection of SARS-Coronavirus-specific B cell epitopes by phage peptide library screening and evaluation of the immunological effect of epitope-based peptides on mice. *Virology* **2007**, *359*, 264-274, doi:10.1016/j.virol.2006.09.016.
43. He, Y.X.; Li, J.J.; Heck, S.; Lustigman, S.; Jiang, S.B. Antigenic and immunogenic characterization of recombinant baculovirus-expressed severe acute respiratory syndrome coronavirus spike protein: Implication for vaccine design. *J Virol* **2006**, *80*, 5757-5767, doi:10.1128/jvi.00083-06.
44. He, Y.X.; Li, J.J.; Du, L.Y.; Yan, X.X.; Hu, G.G.; Zhou, Y.S.; Jiang, S.B. Identification and characterization of novel neutralizing epitopes in the receptor-binding domain of SARS-CoV spike protein: Revealing the critical antigenic determinants in inactivated SARS-CoV vaccine. *Vaccine* **2006**, *24*, 5498-5508, doi:10.1016/j.vaccine.2006.04.054.
45. Yu, X.F.; Liang, L.H.; She, M.; Liao, X.L.; Gu, J.; Li, Y.H.; Han, Z.C. Production of a monoclonal antibody against SARS-CoV spike protein with single intrasplenic immunization of plasmid DNA. *Immunol Lett* **2005**, *100*, 177-181, doi:10.1016/j.imlet.2005.03.015.
46. Hua, R.H.; Zhou, Y.J.; Wang, Y.F.; Hua, Y.Z.; Tong, G.Z. Identification of two antigenic epitopes on SARS-CoV spike protein. *Biochem Biophys Res Commun* **2004**, *319*, 929-935, doi:10.1016/j.bbrc.2004.05.066.
47. Bian, C.; Zhang, X.Q.; Cai, X.F.; Zhang, L.Q.; Chen, Z.W.; Zha, Y.; Xu, Y.; Xu, K.; Lu, W.; Yan, L.C., et al. Conserved amino acids W423 and N424 in receptor-binding domain of SARS-CoV are potential targets for therapeutic monoclonal antibody. *Virology* **2009**, *383*, 39-46, doi:10.1016/j.virol.2008.09.029.

48. Guo, J.P.; Petric, M.; Campbell, W.; McGeer, P.L. SARS corona virus peptides recognized by antibodies in the sera of convalescent cases. *Virology* **2004**, *324*, 251-256, doi:10.1016/j.virol.2004.04.017.
49. Hu, H.B.; Li, L.; Kao, R.Y.; Kou, B.B.; Wang, Z.G.; Zhang, L.; Zhang, H.Y.; Hao, Z.Y.; Tsui, W.H.; Ni, A.P., et al. Screening and identification of linear B-cell epitopes and entry-blocking peptide of severe acute respiratory syndrome (SARS)-associated coronavirus using synthetic overlapping peptide library. *J Comb Chem* **2005**, *7*, 648-656, doi:10.1021/cc0500607.
50. Grifoni, A.; Sidney, J.; Zhang, Y.; Scheuermann, R.H.; Peters, B.; Sette, A. A Sequence Homology and Bioinformatic Approach Can Predict Candidate Targets for Immune Responses to SARS-CoV-2. *Cell Host Microbe* **2020**, *12*, 30166-30169.
51. Lee, J.S.; Poo, H.; Han, D.P.; Hong, S.P.; Kim, K.; Cho, M.W.; Kim, E.; Sung, M.H.; Kim, C.J. Mucosal immunization with surface-displayed severe acute respiratory syndrome coronavirus spike protein on *Lactobacillus casei* induces neutralizing antibodies in mice. *J Virol* **2006**, *80*, 4079-4087, doi:10.1128/jvi.80.8.4079-4087.2006.
52. Greenough, T.C.; Babcock, G.J.; Roberts, A.; Hernandez, H.J.; Thomas, W.D.; Coccia, J.A.; Graziano, R.F.; Srinivasan, M.; Lowy, I.; Finberg, R.W., et al. Development and characterization of a severe acute respiratory syndrome-associated coronavirus-neutralizing human monoclonal antibody that provides effective immunoprophylaxis in mice. *J Infect Dis* **2005**, *191*, 507-514, doi:10.1086/427242.
53. Robson, B. Computers and viral diseases. Preliminary bioinformatics studies on the design of a synthetic vaccine and a preventative peptidomimetic antagonist against the SARS-CoV-2 (2019-nCoV, COVID-19) coronavirus. *Comput Biol Med* **2020**, *119*, 26.
54. Elshabrawy, H.A.; Coughlin, M.M.; Baker, S.C.; Prabhakar, B.S. Human Monoclonal Antibodies against Highly Conserved HR1 and HR2 Domains of the SARS-CoV Spike Protein Are More Broadly Neutralizing. *PLoS One* **2012**, *7*, doi:10.1371/journal.pone.0050366.
55. Pimentel, T.; Yan, Z.; Jeffers, S.A.; Holmes, K.V.; Hodges, R.S.; Burkhard, P. Peptide Nanoparticles as Novel Immunogens: Design and Analysis of a Prototypic Severe Acute Respiratory Syndrome Vaccine. *Chem Biol Drug Des* **2009**, *73*, 53-61, doi:10.1111/j.1747-0285.2008.00746.x.
56. Yu, H.; Jiang, L.F.; Fang, D.Y.; Yan, H.J.; Zhou, J.J.; Zhou, J.M.; Liang, Y.; Gao, Y.; Zhao, W.; Long, B.G. Selection of SARS-coronavirus-specific B cell epitopes by phage peptide library screening and evaluation of the immunological effect of epitope-based peptides on mice. *Virology* **2007**, *359*, 264-274.
57. Chow, S.C.S.; Ho, C.Y.S.; Tam, T.T.Y.; Wu, C.; Cheung, T.; Chan, P.K.S.; Ng, M.H.L.; Hui, P.K.; Ng, H.K.; Au, D.M.Y., et al. Specific epitopes of the structural and hypothetical proteins elicit variable humoral responses in SARS patients. *J Clin Pathol* **2006**, *59*, 468-476, doi:10.1136/jcp.2005.029868.
58. Lip, K.M.; Shen, S.; Yang, X.M.; Keng, C.T.; Zhang, A.H.; Oh, H.L.J.; Li, Z.H.; Hwang, L.A.; Chou, C.F.; Fielding, B.C., et al. Monoclonal antibodies targeting the HR2 domain and the region immediately upstream of the HR2 of the S protein

neutralize in vitro infection of severe acute respiratory syndrome coronavirus. *J Virol* **2006**, *80*, 941-950, doi:10.1128/jvi.80.2.941-950.2006.
59. Lai, S.C.; Chong, P.C.S.; Yeh, C.T.; Liu, L.S.J.; Jan, J.T.; Chi, H.Y.; Liu, H.W.; Chen, A.; Wang, Y.C. Characterization of neutralizing monoclonal antibodies recognizing a 15-residues epitope on the spike protein HR2 region of severe acute respiratory syndrome coronavirus (SARS-CoV). *J Biomed Sci* **2005**, *12*, 711-727, doi:10.1007/s11373-005-9004-3.
60. Hua, R.H.; Wang, Y.F.; Bu, Z.G.; Zhou, Y.J.; Ge, J.Y.; Wang, X.J.; Tong, G.Z. Identification and antigenic epitope mapping of immunodominant region amino residues 510 to 672 on the spike protein of the severe acute respiratory syndrome coronavirus. *DNA Cell Biol* **2005**, *24*, 503-509, doi:10.1089/dna.2005.24.503.
61. Lin, Y.S.; Lin, C.F.; Fang, Y.T.; Kuo, Y.M.; Liao, P.C.; Yeh, T.M.; Hwa, K.Y.; Shieh, C.C.K.; Yen, J.H.; Wang, H.J., et al. Antibody to severe acute respiratory syndrome (SARS)-associated coronavirus spike protein domain 2 cross-reacts with lung epithelial cells and causes cytotoxicity. *Clin Exp Immunol* **2005**, *141*, 500-508, doi:10.1111/j.1365-2249.2005.02864.x.
62. Lu, W.; Wu, X.D.; De Shi, M.; Yang, R.F.; He, Y.Y.; Bian, C.; Shi, T.L.; Yang, S.; Zhu, X.L.; Jiang, W.H., et al. Synthetic peptides derived from SARS coronavirus S protein with diagnostic and therapeutic potential. *FEBS Lett* **2005**, *579*, 2130-2136, doi:10.1016/j.febslet.2005.02.070.
63. Zhong, X.F.; Yang, H.H.; Guo, Z.F.; Sin, W.Y.F.; Chen, W.; Xu, J.J.; Fu, L.; Wu, J.; Mak, C.K.G.; Cheng, C.S.S., et al. B-cell responses in patients who have recovered from severe acute respiratory syndrome target a dominant site in the S2 domain of the surface spike glycoprotein. *J Virol* **2005**, *79*, 3401-3408, doi:10.1128/jvi.79.6.3401-3408.2005.
64. Ren, Y.; Zhou, Z.; Liu, J.; Lin, L.; Li, S.; Wang, H.; Xia, J.; Zhao, Z.; Wen, J.; Zhou, C., et al. A strategy for searching antigenic regions in the SARS-CoV spike protein. *Genom Proteom Bioinf* **2003**, *1*, 207-215.
65. Zhou, T.; Wang, H.; Luo, D.L.; Rowe, T.; Wang, Z.; Hogan, R.J.; Qiu, S.H.; Bunzel, R.J.; Huang, G.Q.; Mishra, V., et al. An exposed domain in the severe acute respiratory syndrome coronavirus spike protein induces neutralizing antibodies. *J Virol* **2004**, *78*, 7217-7226, doi:10.1128/jvi.78.13.7217-7226.2004.
66. Wang, J.Q.; Wen, J.; Li, J.X.; Yin, J.N.; Zhu, Q.Y.; Wang, H.; Yang, Y.K.; De Qin, E.; You, B.; Li, W., et al. Assessment of immunoreactive synthetic peptides from the structural proteins of severe acute respiratory syndrome coronavirus. *Clin Chem* **2003**, *49*, 1989-1996, doi:10.1373/clinchem.2003.023184.
67. Wang, Q.D.; Zhang, L.F.; Kuwahara, K.; Li, L.; Liu, Z.J.; Li, T.S.; Zhu, H.; Liu, J.N.; Xu, Y.F.; Xie, J., et al. Immunodominant SARS Coronavirus Epitopes in Humans Elicited both Enhancing and Neutralizing Effects on Infection in Non-human Primates. *ACS Infect Dis* **2016**, *2*, 361-376, doi:10.1021/acsinfecdis.6b00006.
68. Miyoshi-Akiyama, T.; Ishida, I.; Fukushi, M.; Yamaguchi, K.; Matsuoka, Y.; Ishihara, T.; Tsukahara, M.; Hatakeyama, S.; Itoh, N.; Morisawa, A., et al. Fully Human Monoclonal Antibody Directed to Proteolytic Cleavage Site in Severe Acute Respiratory Syndrome (SARS) Coronavirus S Protein Neutralizes the Virus in a Rhesus Macaque SARS Model. *J Infect Dis* **2011**, *203*, 1574-1581, doi:10.1093/infdis/jir084.

69. Shichijo, S.; Keicho, N.; Long, H.T.; Quy, T.; Phi, N.C.; Ha, L.D.; Ban, V.V.; Itoyama, S.; Hu, C.J.; Komatsu, N., et al. Assessment of synthetic peptides of severe acute respiratory syndrome coronavirus recognized by long-lasting immunity. *Tissue Antigens* **2004**, *64*, 600-607, doi:10.1111/j.1399-0039.2004.00314.x.
70. Ng, O.W.; Keng, C.T.; Leung, C.S.W.; Peiris, J.S.M.; Poon, L.L.M.; Tan, Y.J. Substitution at Aspartic Acid 1128 in the SARS Coronavirus Spike Glycoprotein Mediates Escape from a S2 Domain-Targeting Neutralizing Monoclonal Antibody. *PLoS One* **2014**, *9*, doi:10.1371/journal.pone.0102415.
71. Qian, C.; Qin, D.; Tang, Q.; Zeng, Y.; Tang, G.X.; Lu, C. Identification of a B-cell antigenic epitope at the N-terminus of SARS-CoV M protein and characterization of monoclonal antibody against the protein. *Virus Genes* **2006**, *33*, 147-156, doi:10.1007/s11262-005-0050-8.
72. Yu, M.; Stevens, V.; Berry, J.D.; Crameri, G.; McEachern, J.; Tu, C.C.; Shi, Z.L.; Liang, G.D.; Weingartl, H.; Cardosa, J., et al. Determination and application of immunodominant regions of SARS coronavirus spike and nucleocapsid proteins recognized by sera from different animal species. *J Immunol Methods* **2008**, *331*, 1-12, doi:10.1016/j.jim.2007.11.009.
73. van den Brink, E.N.; ter Meulen, J.; Cox, F.; Jongeneelen, M.A.C.; Thijsse, A.; Throsby, M.; Marissen, W.E.; Rood, P.M.L.; Bakker, A.B.H.; Gelderblom, H.R., et al. Molecular and biological characterization of human monoclonal antibodies binding to the spike and nucleocapsid proteins of severe acute respiratory syndrome coronavirus. *J Virol* **2005**, *79*, 1635-1644, doi:10.1128/jvi.79.3.1635-1644.2005.
74. Shin, G.C.; Chung, Y.S.; Kim, I.S.; Cho, H.W.; Kang, C. Preparation and characterization of a novel monoclonal antibody specific to severe acute respiratory syndrome-coronavirus nucleocapsid protein. *Virus Res* **2006**, *122*, 109-118, doi:10.1016/j.virusres.2006.07.004.
75. Bussmann, B.M.; Reiche, S.; Jacob, L.H.; Braun, J.M.; Jassoy, C. Antigenic and cellular localisation analysis of the severe acute respiratory syndrome coronavirus nucleocapsid protein using monoclonal antibodies. *Virus Res* **2006**, *122*, 119-126, doi:10.1016/j.virusres.2006.07.005.
76. Liu, S.J.; Leng, C.H.; Lien, S.P.; Chi, H.Y.; Huang, C.Y.; Lin, C.L.; Lian, W.C.; Chen, C.J.; Hsieh, S.L.; Chong, P. Immunological characterizations of the nucleocapsid protein based SARS vaccine candidates. *Vaccine* **2006**, *24*, 3100-3108, doi:10.1016/j.vaccine.2006.01.058.
77. Liang, Y.F.; Wan, Y.; Qiu, L.W.; Zhou, J.G.; Ni, B.; Guo, B.; Zou, Q.; Zou, L.Y.; Zhou, W.; Jia, Z.C., et al. Comprehensive antibody epitope mapping of the nucleocapsid protein of severe acute respiratory syndrome (SARS) coronavirus: Insight into the humoral immunity of SARS. *Clin Chem* **2005**, *51*, 1382-1396, doi:10.1373/clinchem.2005.051045.
78. Tang, T.K.; Wu, M.P.J.; Chen, S.T.; Hou, M.H.; Hong, M.H.; Pan, F.M.; Yu, H.M.; Chen, J.H.; Yao, C.W.; Wang, A.H.J. Biochemical and immunological studies of nucleocapsid proteins of severe acute respiratory syndrome and 229E human coronaviruses. *Proteomics* **2005**, *5*, 925-937, doi:10.1002/pmic.200401204.
79. Li, S.; Lin, L.; Wang, H.; Yin, J.; Ren, Y.; Zhao, Z.; Wen, J.; Zhou, C.; Zhang, X.; Li, X., et al. The epitope study on the SARS-CoV nucleocapsid protein. *Genom Proteom Bioinf* **2003**, *1*, 198-206.

80. He, Y.X.; Zhou, Y.S.; Wu, H.; Kou, Z.H.; Liu, S.W.; Jiang, S.B. Mapping of antigenic sites on the nucleocapsid protein of the severe acute respiratory syndrome coronavirus. *J Clin Microbiol* **2004**, *42*, 5309-5314, doi:10.1128/jcm.42.11.5309-5314.2004.
